# Effect of CHST11, a novel biomarker, on the biological functionalities of clear cell renal cell carcinoma

**DOI:** 10.1038/s41598-024-58280-8

**Published:** 2024-04-02

**Authors:** Weijing Hu, Yongquan Chen, Lin Zhang, Xiaoling Guo, Xin Wei, Yuan Shao, Dongwen Wang, Bo Wu

**Affiliations:** 1 Department of Urology, First Hospital of Shanxi Medical University, Taiyuan, 030001 Shanxi China; 2 Department of Urology, Shanxi Coal Center Hospital, Taiyuan, 030001 Shanxi China; 3 Shanxi Medical University, Taiyuan, 030001 Shanxi China; 4 Geriatrics Department, Xi’an Central Hospital, Xi’an, 710003 China; 5 Department of Urology, The Second Hospital of Tianjin Medical University, Tianjin, 300070 China; 6 National Cancer Center/National Clinical Research Center for Cancer/Cancer Hospital and Shenzhen Hospital, Chinese Academy of Medical Sciences and Peking Union Medical College, Shenzhen, 518116 Guangdong China

**Keywords:** Clear cell renal cell carcinoma, Carbohydrate sulfotransferase 11, Proliferation, Migration, Invasion, Biomarker, Tumor microenvironment, Computational biology and bioinformatics, Cancer, Urological cancer

## Abstract

Clear cell renal cell carcinoma (ccRCC) is a common malignant tumor, and the role of carbohydrate sulfotransferase 11 (CHST11) in this cancer remains unclear. Here, by using bioinformatics methods, we comprehensively analyzed the relationship between CHST11 and clinical significance, immune infiltration, functional enrichment, m^6^A methylation, and protein–protein interaction networks. We found that CHST11 expression was significantly higher in ccRCC samples than in normal tissues. Additionally, CHST11 levels correlated with the clinicopathological features of ccRCC patients and functioned as a prognostic factor for patient survival. Functional analysis revealed the involvement of CHST11 in metabolic pathways. Immune infiltration and m^6^A methylation analysis suggested the association of CHST11 with immune cell abundance in the tumor microenvironment and specific methylation patterns in ccRCC. The in vitro analysis of the clinical samples and ccRCC cell lines demonstrated that the overexpression of CHST11 promotes ccRCC cell proliferation, migration, and invasion, while its suppression has the opposite effect. Thus, CHST11 may play a remarkable role in the occurrence and progression of ccRCC. Functionally, CHST11 promotes the aggressiveness of ccRCC cells. These findings provide insights into the role of CHST11 in ccRCC progression.

Registry and the Registration No. of the study/trial: No. 2021K034.

## Introduction

In recent years, the global incidence of renal cell carcinoma (RCC) has steadily increased. Clear cell renal cell carcinoma (ccRCC) is the predominant type of RCC and accounts for approximately 80% of RCC cases^1^. Radical nephrectomy remains the primary treatment approach for ccRCC in clinical practice. In the past decade, the widespread application of novel biological agents and other technologies has improved the treatment efficacy for advanced renal cancer. However, given the mild early clinical symptoms of ccRCC, metastasis often occurs by the time the tumor is diagnosed. Consequently, the overall survival (OS) rate of patients with metastatic RCC is < 10% at 5 years after diagnosis^2^. Hence, it is crucial to explore novel treatment strategies for patients with advanced stage RCC and improve the OS rate.

The tumor metastasis process is intricate and involves several factors and alterations at the molecular level. Chondroitin sulfate (CS) is a member of glycosaminoglycans (GAGs) and performs critical physiological functions. GAGs show various biological effects and play a pivotal role in the interconnection of tumor cells. CS is significantly involved in processes such as cell proliferation, differentiation, and migration and even influences the biological behavior of cancer cells by participating in the activation of cancer-related signaling pathways^3^.

CHST11, also known as chondroitin-4-*O*-sulfotransferase-1 (C4ST-1), is located on chromosome 12q23.3. It plays a critical role in mediating the sulfation reaction of *N*-acetyl galactosamine (GalNAc) and serves as a crucial regulatory enzyme in CS synthesis^4^. Recent studies have indicated that CHST11 is associated with the development of several types of cancer. The elevated expression of CHST11 in patients with lung cancer and pancreatic cancer is correlated with poor prognosis^5,6^. The aberrant expression of CHST11 facilitates the proliferation, migration, and invasion of tumor cells and promotes the emergence of biological behavior characteristics of cancer stem cells^7,8^. However, the role of the *CHST11* gene in ccRCC remains uncertain.

In the present study, we investigated the differential expression of the *CHST11* gene between ccRCC tissues and normal tissues, its association with clinicopathological characteristics, and its prognostic relevance. We performed immune infiltration analysis, functional enrichment analysis, m^6^A methylation analysis, and protein–protein interaction (PPI) network analysis by using relevant databases. Finally, we validated the mRNA and protein expression levels of CHST11 in ccRCC and adjacent normal tissues by qRT-PCR and immunohistochemistry (IHC) experiments. We also assessed the effect of overexpression or under-expression of the *CHST11* gene in ccRCC cell lines. This comprehensive approach could help establish the role of CHST11 as a prognostic biomarker and potential therapeutic target in ccRCC.

## Materials and methods

### Collection of clinical specimens

Tissue samples were collected from patients who underwent radical nephrectomy in the Department of Urology at the First Hospital of Shanxi Medical University from January 2021 to October 2021. The patients were pathologically diagnosed to have ccRCC after surgery, and both cancerous tissues and adjacent normal tissues were included as research samples. The exclusion criteria were as follows: (1) preoperative exposure to antineoplastic therapies such as chemotherapy, radiotherapy, or molecular targeted therapy; (2) absence of a signed informed consent form; and (3) lack of comprehensive clinical and pathological data. Fifty specimens were included in this study. The study was conducted in accordance with the Declaration of Helsinki (as revised in 2013). The study was approved by the Ethics Committee of the First Hospital of Shanxi Medical University (No. 2021K034), and informed consent was obtained from all the patients.

### Determination of the CHST11 gene expression levels from the database

Datasets were obtained from The Cancer Genome Atlas (TCGA) database^9^. Gene Expression Omnibus (GEO) dataset was downloaded from the NCBI GEO database. The following datasets were included in this study: GSE53757, GSE40435, GSE15641, and GSE36895 (Table S1).

The HPA database^10^ was used to perform an IHC analysis of CHST11 protein expression. We used the program AlphaFoldDB^11^ to predict the three-dimensional structure of the *CHST11* gene. Receiver operating characteristic (ROC) curve analysis was conducted on the TCGA-KIRC, GSE53757, GSE40435, GSE15641, and GSE36895 datasets. The area under the curve (AUC) is a commonly used metric to assess diagnostic tests.

### Clinicopathological analysis of CHST11 and its prognostic value

We used the TCGA-KIRC dataset to estimate the prognostic value of CHST11 in ccRCC patients. Kaplan–Meier (KM) survival curves were generated using the survival package, and log-rank tests were performed. The prognostic outcomes were OS, disease-specific survival (DSS), and progression-free interval (PFI).

### Immune infiltration analysis

The Tumor Immune Estimation Resource (TIMER)^12^ was used to investigate the correlation between the *CHST11* gene expression and immune infiltration levels in ccRCC patients. KM Plotter^13^ was utilized for further analysis to determine whether CHST11 expression affects the prognosis of ccRCC patients following immune cell infiltration. Additionally, single-sample Gene Set Enrichment Analysis (ssGSEA) was used to quantitatively assess the correlation between the infiltration levels of 24 types of immune cells and CHST11 expression^14^. The Tumor and Immune System Interaction Database (TISIDB) was used to examine the relationship between CHST11 expression and tumor lymphocyte infiltration, immune suppressors, immune stimulators, and major histocompatibility complex. Tumor Immune Single-cell Hub 2 (TISCH2)^15^ was used to demonstrate the relationship between the colocalized expression of CHST11 and immune cells at the single-cell RNA sequencing level.

### m^6^A methylation analysis of CHST11

Enzymes involved in m^6^A methylation include methyltransferases (writers), demethylases (erasers), and methylation readers (readers). We conducted a study to investigate the relationship between CHST11 expression and the aforementioned 23 types of m^6^A methylation regulators. We used LASSO regression to calculate a risk score and established a model. The patients were stratified into high-risk and low-risk groups, and the KM survival analysis was conducted to assess the prognostic value. ROC curves were used to analyze the sensitivity and specificity of the model. We then obtained the relevant methylation data for ccRCC from the Xena database^16^. We finally identified the core methylation regions associated with patient prognosis.

### PPI and enrichment analysis

We used the STRING database^17^ to generate a PPI network of proteins associated with CHST11. We then conducted a comprehensive analysis of CHST11 and its associated genes by Gene Ontology (GO) analysis and the Kyoto Encyclopedia of Genes and Genomes (KEGG) pathway enrichment analysis, including GO biological process (BP), cellular constituent (CC), molecular function (MF), and KEGG pathway functional enrichment analysis.

### Quantitative real-time polymerase chain reaction

The CHST11 mRNA expression was assessed in 50 pairs of ccRCC tissues and adjacent noncancerous tissues. Total RNA was extracted using TRIzol (Transgene, Beijing, China) reagent, followed by the synthesis of the first-strand cDNA using the Uni All-in-One SuperMix mRNA reverse transcription kit (Transgene, Beijing, China). The obtained cDNA was then subjected to qPCR using the PerfectStart Green qPCR SuperMix reagent kit (Transgene, Beijing, China). GAPDH was used as the internal reference. The primer sequences were as follows: GAPDH-F: 5′-GCTCTCTGCTCCTCCTGTTC-3′; GAPDH-R: 5′-ACGACCCHST11TCCGTTGACTC-3′. For CHST11, the primer sequences were as follows: CHST11-F: 5′-CACAAGCCGTAAGCGGAGG-3′; CHST11-R: 5′-CATGGGGTCGCTGTACTTCC-3′. The relative gene expression levels were calculated using the 2^−ΔΔCt^ method.

### IHC analysis

IHC was used to evaluate the CHST11 protein expression levels in 50 paired sets of ccRCC tissues and the corresponding adjacent normal tissues. Tissue sections of pathological origin were obtained from patients with confirmed ccRCC through pathological assessment. These sections were subjected to deparaffinization, hydration, and removal of endogenous peroxidase with hydrogen peroxide to retrieve tissue antigens. The sections were then washed thrice with PBS for 3 min each. The sections were then incubated with anti-CHST11 antibodies (1:300, ZSGB-BIO, Chengdu, China) at 37 °C for 1.5 h, followed by cooling at room temperature for 35 min. Following a rinse in PBS, secondary antibodies (Boster, Wuhan, China) were added, and the sections were incubated for 30 min. Another round of rinsing in PBS was performed before using the DAB kit (Boster, Wuhan, China) for color development; the reaction was terminated after microscopic observation. Hematoxylin was used for counterstaining acidophilic structures, which produced a blue hue. The sections were subsequently dehydrated, treated for transparency, and mounted with neutral resin. The stained sections were then examined by pathologists.

### Cell culture and transfection

Human renal epithelial normal cells (293T) and human ccRCC cells (786O, 769P, and ACHN) were procured from the Cell Bank of the Shanghai Institute of Life Sciences. The cells were cultured in Dulbecco’s Modified Eagle’s Medium (DMEM) (Procell, Wuhan, China) supplemented with 10% fetal bovine serum (FBS) (Gibco, CA, USA) and 1% penicillin/streptomycin (Solarbio, Beijing, China). For transfection, the overexpression plasmid and knockdown small interfering RNA (siRNA) were obtained from Han-Bio (Shanghai, China). In accordance with the manufacturer’s instructions, transfection was performed using Lipofectamine 3000 (Thermo Fisher Scientific, CA, USA) for plasmids or siRNAs. Subsequent experiments were conducted 48 h post-transfection.

### Cell counting kit-8 assay

After cell digestion with trypsin (Solarbio, Beijing, China), the cell suspension was adjusted to a concentration of 5 × 10^3^ cells/100 µL/well and added to a 96-well plate (Solarbio). Next, 100 µL of the prepared CCK-8 reagent (MCE, NJ, USA) was added to each well. The absorbance values were measured using the microplate reader at 0, 24, 48, 72, and 96 h after cell culture. The absorbance was measured at 450 nm by using a microplate reader.

### Wound healing assay

After the cells covered the entire 6-well plate, vertical scratches were made at the bottom of the plate by using a sterile 200 µL pipette tip. The cell debris was washed with PBS (Solarbio) three times. A basic culture medium without FBS was added to continue cell cultivation. At 0, 12, 24, and 48 h after cell growth, photographs were captured under a microscope. The area of wound healing was determined using the ImageJ software.

### Transwell assay

The cells were digested with trypsin (Solarbio), neutralized with an FBS-free medium, and resuspended after centrifugation to prepare a cell suspension. After mixing the Matrigel matrix (Corning, NY, USA) with a basic culture medium, the mixture was added (or not added) to the Transwell chamber and incubated for 3 h. Next, the cell density was adjusted to 3 × 10^5^ cells/mL, and the cells were then added to the Transwell chamber. The cells were cultured in a cell culture incubator at 37 °C for 24 h. The cells were fixed with paraformaldehyde, stained with crystal violet, and observed and counted under a microscope.

### Statistical analysis

All data were analyzed using GraphPad Prism 9.0 software and R software (version 4.2.0). Student’s t-test was used to compare two groups, and one-way ANOVA was used to compare multiple groups. Survival analysis was conducted using KM curves, along with the determination of hazard ratio (HR) and *p*-value through the log-rank test with 95% confidence intervals. Pearson’s or Spearman’s correlation analyses were used to assess the correlation between genes. Differences at the *P*-value of < 0.05 were considered statistically significant.

### Ethical approval

Approval of the research protocol by an Institutional Reviewer Board:the Ethics Committee of the First Hospital of Shanxi Medical University.

### Informed consent

Informed consent was taken from all the patients.

## Results

### *CHST11* gene expression level in ccRCC tissues

**Figure 1:**
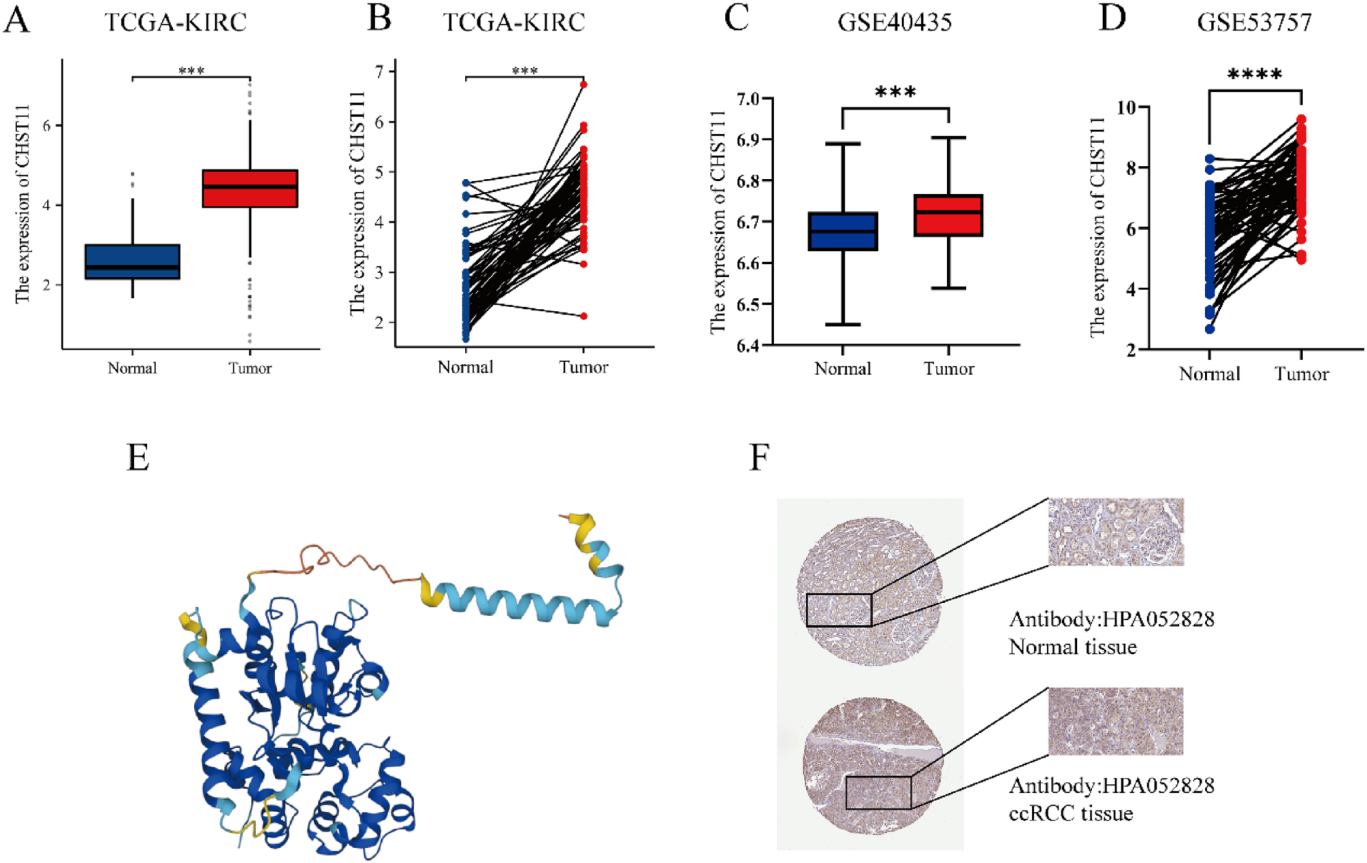
Expression of CHST11 at the mRNA and protein levels in online databases. (**A**) Expression of CHST11 in nonpaired tissues. (**B**) Expression of CHST11 in paired tissues. (**C**) Expression of CHST11 in the GSE40435 dataset. (**D**) Expression of CHST11 in the GSE53757 dataset. (**E**) Three-dimensional structure of CHST11. (**F**) IHC results of CHST11 in tumor and normal tissues (***P < 0.001, ****P < 0.0001).

Following the analysis of the downloaded TCGA-KIRC datasets, the results indicated that the *CHST11* gene expression level was higher in tumor tissues (n = 541) than in normal tissues (n = 72) (Fig. 1A). Regarding paired tissues, the *CHST11* gene expression level was higher in tumor tissues (n = 72) than in normal tissues (n = 72); these findings were consistent with those of nonpaired tissues (*P* < 0.001) (Fig. 1B). The analysis of mRNA expression on the gene chips GSE40435 and GSE53757 showed that the *CHST11* gene was overexpressed in ccRCC (Fig. 1C,D).

We then examined CHST11 protein expression levels in ccRCC and normal tissues by using the HPA database (Fig. 1F). The three-dimensional structure was predicted using the AlphaFold database, as shown in Fig. 1E.

### Relationship between CHST11 expression levels and clinicopathological features in ccRCC patients

**Figure 2:**
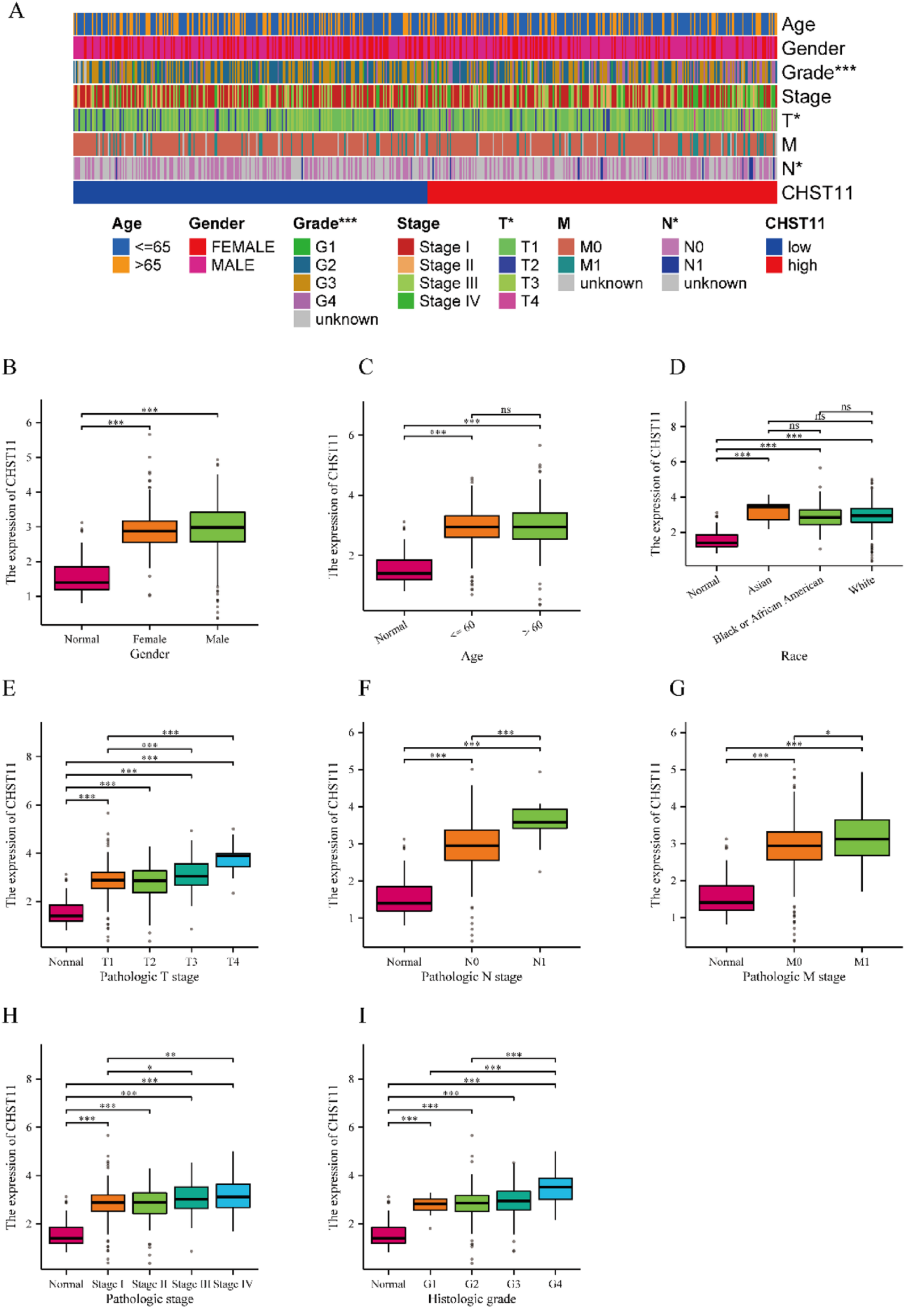
The relationship between CHST11 mRNA expression level and clinical characteristics in the TCGA database. (**A**) Heatmap. Association between the CHST11 mRNA expression level and gender (**B**), age (**C**), ethnicity (**D**), T staging (**E**), N staging (**F**), M staging (**G**), AJCC staging (**H**), and WHO/ISUP histological grading (**I**) (*P < 0.05, **P < 0.01, ***P < 0.001, ****P < 0.0001) (This heatmap was generated by R software version 4.2.0, https://cloud.r-project.org/).

A total of 541 patients were included in the present study. The CHST11 mRNA expression level showed significant correlations with T-stage, N-stage, M-stage, AJCC staging, and WHO/ISUP-histologic grade. However, no correlation was observed between the CHST11 mRNA expression level and gender, age, or race (Table S2) (Fig. 2). The logistic regression analysis indicated a positive correlation between the CHST11 mRNA expression level and T-stage, N-stage, AJCC staging, and WHO/ISUP-histologic grade (Table S3).

### Diagnostic efficacy and prognostic value of CHST11 in ccRCC

**Figure 3:**
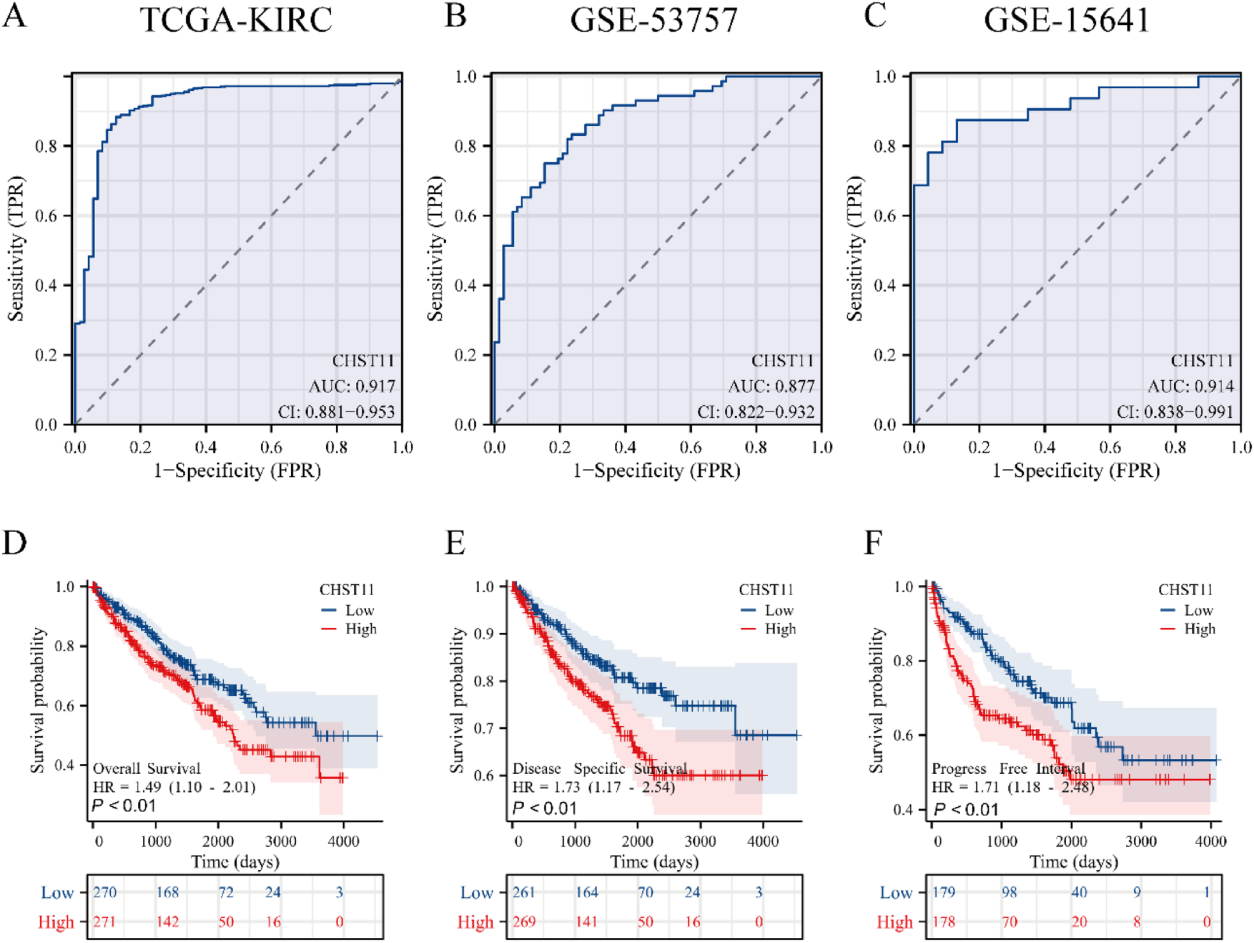
Diagnostic efficacy and survival prognosis of CHST11 in ccRCC. ROC curves of CHST11 for diagnosing ccRCC in the TCGA (**A**), GSE53757 (**B**), and GSE15641 (**C**) datasets. Correlation between CHST11 expression and OS (**D**), DSS (**E**), and PFI (**F**). (OS, overall survival; DSS, disease-specific survival; PFI, progression-free interval).

We used ROC curves and AUC values to assess the diagnostic efficacy of CHST11 in ccRCC. The KM plotter was used to analyze the survival outcomes associated with CHST11 in ccRCC. The results indicated that the AUC values were 0.917, 0.877, and 0.914 in the TCGA-KIRC, GSE53757, and GSE15641 datasets, respectively (Fig. 3A–C). Moreover, the AUC values exceeded 0.6 in both GSE36895 and GSE40435 datasets (Fig. S1). KM survival analysis revealed a significant correlation between the high CHST11 expression and poor OS, DSS, and PFI (Fig. 3D–F).

### Correlation between CHST11 and immune cell infiltration in ccRCC

We used the TIMER database, ssGSEA, and TISIDB database to analyze the correlation between CHST11 expression levels and immune responses. The KM plotter database was used to conduct an in-depth analysis of the relationship between immune cell infiltration and prognosis of ccRCC patients. Finally, we used the TISCH2 database for single-cell RNA sequencing to provide information on the co-localization of immune cell infiltration.

The TIMER database revealed a close positive correlation between CHST11 expression and six immune cell types: B cells, CD8^+^ T cells, CD4^+^ T cells, macrophages, neutrophils, and dendritic cells (Fig. S2A). Additionally, CHST11 expression showed a positive correlation with the expression of six immune checkpoint genes: *HAVCR2*, *CD274*, *CTLA4*, *PDCD1*, *LAG3*, and *PDCD1LG2* (Fig. S2B). The survival analysis module in the database, however, indicated that the infiltration of these six immune cell types was not significantly associated with the OS of ccRCC patients (Fig. S2C).

The results of ssGSEA indicated that CHST11 exhibited significant differences in immune cell infiltration levels between the high and low expression groups in the majority of somatic cells (Fig. S3A). The CHST11 expression levels showed a significant positive correlation with the enrichment of macrophages, T helper cell 1, and T helper cell 2 and exhibited a significant negative correlation with T helper cell 17 enrichment (Fig. S3B).

By using the TISIDB database, Spearman’s correlation analysis was performed between the CHST11 expression level and tumor lymphocytes, tumor immunostimulators, tumor immunoinhibitors, and major histocompatibility complex (MHC) proteins. The results showed a significant correlation between CHST11 and certain tumor immunostimulators (CD28, IL2RA, and TNFSF13B), tumor immunoinhibitors (CD96 and LGALS9), and MHC proteins (HLA-DRA and HLA-DMB) (Tables S4–S7).

**Figure 4:**
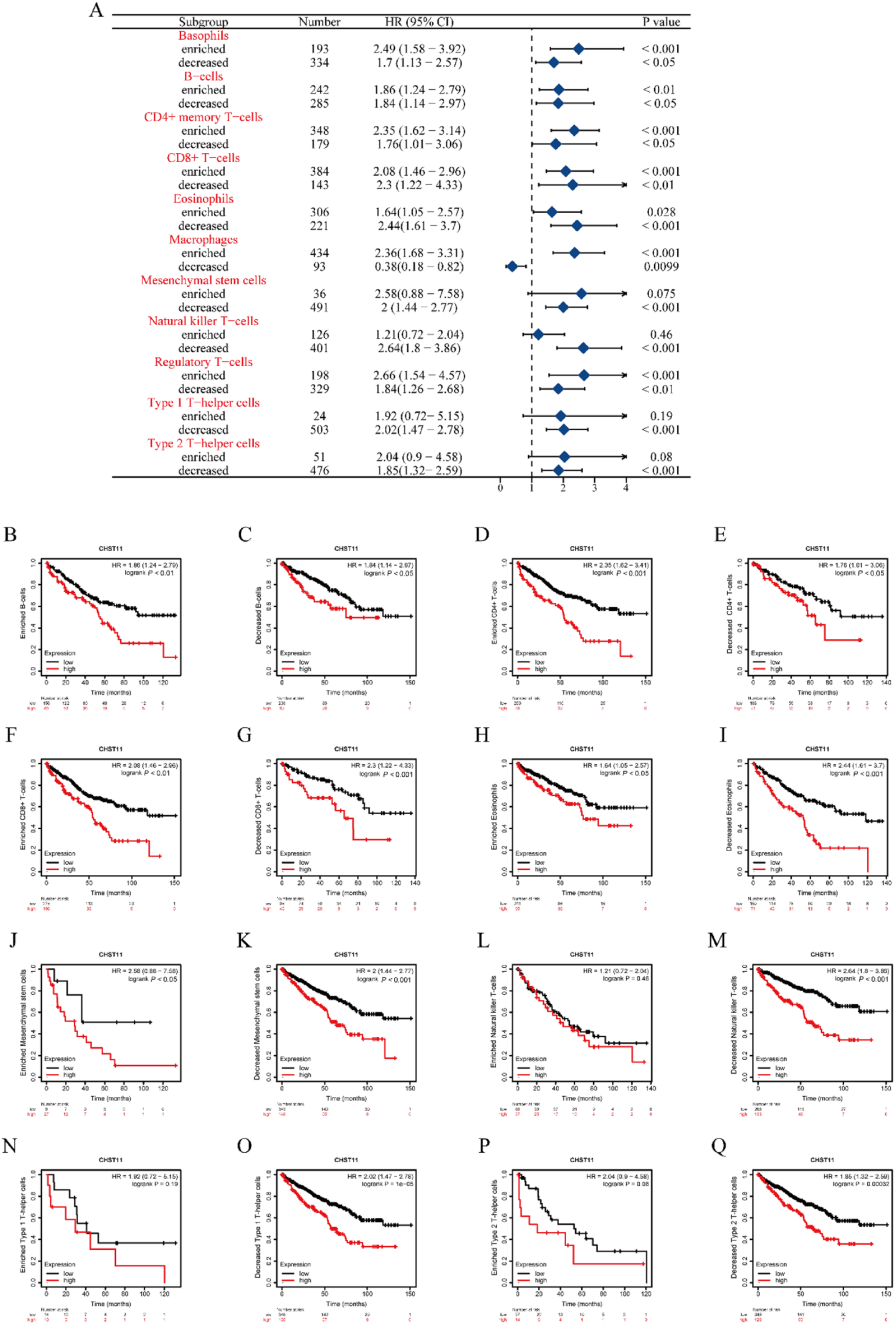
Correlation between CHST11 expression and different immune cell subsets in ccRCC in the Kaplan–Meier (KM) plotter database. (**A**) Forest plot. (**B**–**Q**) Correlation between high CHST11 expression and prognosis of ccRCC patients in different immune cell subsets.

We also conducted a detailed investigation using the KM plotter database and confirmed that ccRCC patients with high CHST11 expression exhibited an increase in macrophage infiltration, along with a decrease in the infiltration of mesenchymal stem cells, natural killer (NK) T cells, type 1 T-helper cells, and type 2 T-helper cells, which correlated with poor patient prognosis (Fig. 4A–Q).

**Figure 5:**
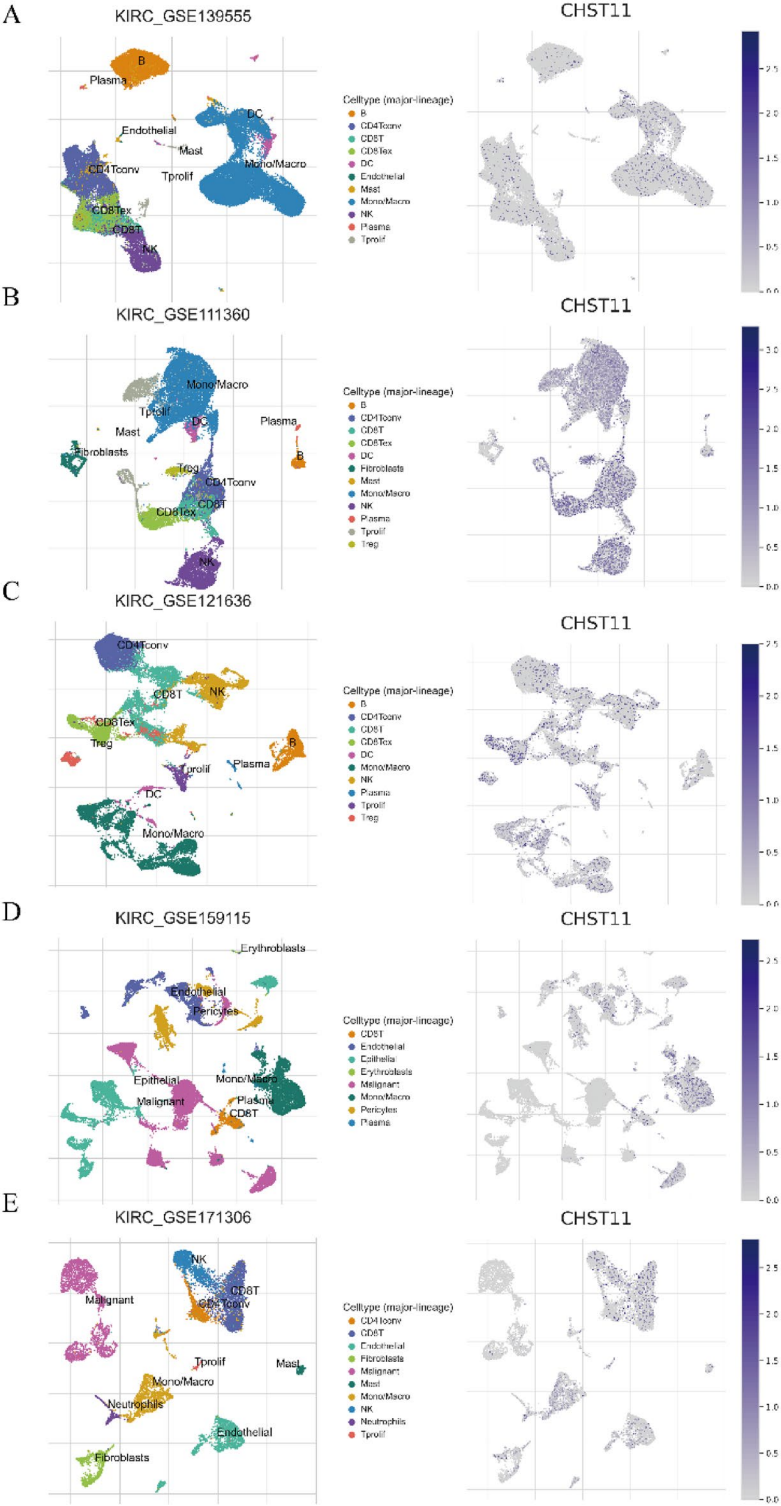
Co-localization of CHST11 expression with immune cell expression in the TISCH2 database. Co-localization heatmap from the GSE139555 (**A**), GSE111360 (**B**), GSE121636 (**C**), GSE159115 (**D**), and GSE171306 (**E**) datasets.

Finally, we conducted an analysis at the single-cell sequencing level with a focus on cell types and the TME in ccRCC. The result was visually represented in the form of a heatmap to demonstrate co-localization. We also analyzed the correlation between CHST11 expression and immune infiltration abundance (Fig. 5A–E). In the GSE111360, GSE139555, GSE121636, GSE159115, and GSE171306 datasets, CHST11 was predominantly distributed in macrophages, CD4^+^ T cells, CD8^+^ T cells, B cells, and NK cells.

### m^6^A methylation analysis of the ***CHST11*** gene

**Figure 6:**
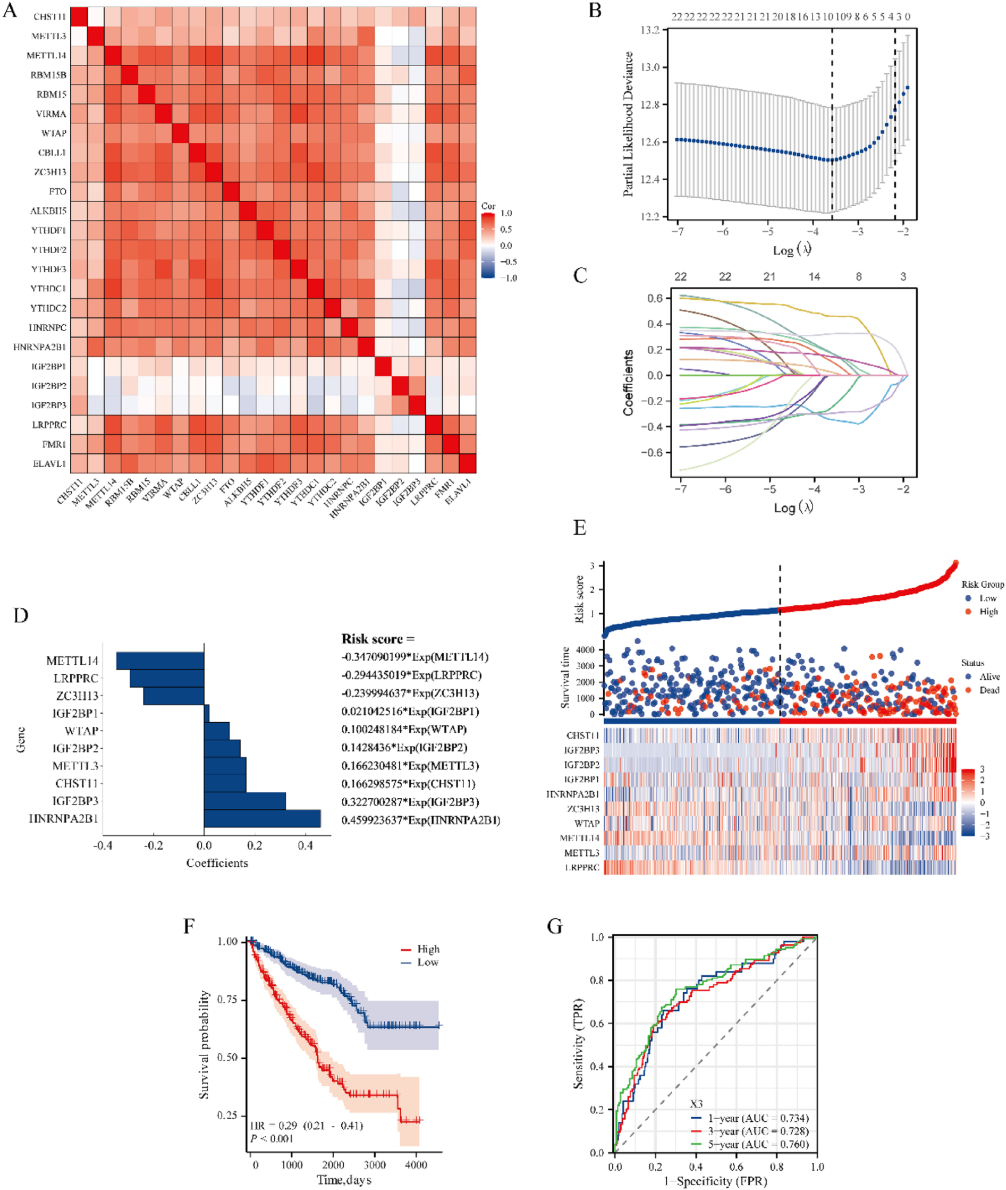
(**A**) Correlation heatmap between CHST11 and 23 m^6^A regulators in ccRCC. (**B**) The LASSO coefficient curve for m^6^A regulators with the minimal λ value determined by tenfold cross-validation in LASSO regression. (**C**) Diagnostic LASSO variable trajectory. (**D**) Correlated genes incorporated into the model and the risk scoring formula. (**E**) Risk factor chart along with the distribution of survival status in independent individuals. (**F**) KM curves for ccRCC patients in the high-risk and low-risk groups. (**G**) ROC curve for the prognostic model (This heatmap was generated by R software version 4.2.0, https://cloud.r-project.org/).

We initially investigated the 23 associated m^6^A methylation genes of CHST11 in ccRCC. The results showed a significant positive correlation between *CHST11* and *RBM15B*, *VIRMA*, *ZC3H13*, *YTHDF3*, *YTHDC2*, and *IGF2BP3* (Fig. 6A). We also conducted LASSO regression analysis with tenfold cross-validation and determined the inclusion genes and the corresponding λ coefficient values for the scoring formula (Fig. 6B,C). We then established a prognostic model and computed the risk scores for each ccRCC patient on the basis of the 10 genes (Fig. 6D,E). Survival analysis indicated that patients in the high-risk group have a poor prognosis in terms of OS (Fig. 6F). The ROC curves calculated for 1, 3, and 5 years of survival displayed high AUC values, thus indicating a favorable predictive accuracy of this model (Fig. 6G).

Subsequently, we downloaded and analyzed the relevant methylation data of TCGA-KIRC from the Xena database. The results indicated that almost all CpG islands were significantly hypermethylated in ccRCC tissues (Fig. S4A). Correlation analysis with CHST11 expression showed a positive association between the methylation status of 31 regions and the CHST11 mRNA expression level (Fig. S4B). We found that only higher methylation levels of cg24946597 were associated with a poor patient prognosis; this finding suggests that cg24946597 is a core methylation region within the *CHST11* gene (Fig. S4C).

### PPI and enrichment analyses of CHST11

**Figure 7:**
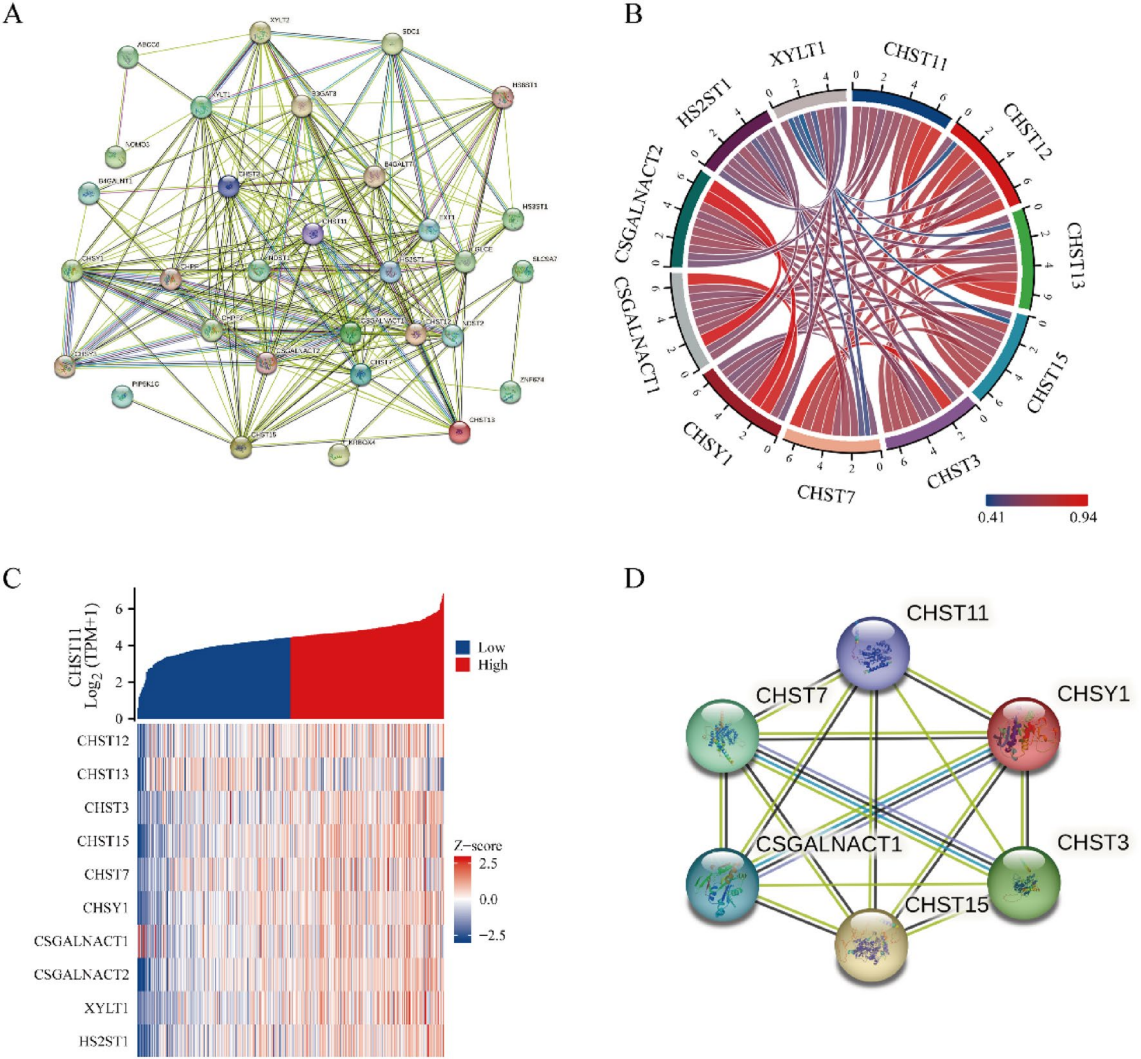
PPI network of CHST11 in ccRCC. (**A**) Interaction network constructed using the STRING database. (**B**) The top 10 genes most closely associated with CHST11. (**C**) Heatmap of the top 10 genes most relevant to CHST11. (**D**) PPI core network of CHST11 (This heatmap was generated by R software version 4.2.0, https://cloud.r-project.org/).

First, we generated a PPI network related to CHST11 by using the STRING database. This network contained 20 proteins, including CHST11 (Fig. 7A). Subsequently, we curated the top 10 genes most closely associated with CHST11. We then constructed an interaction chord diagram that illustrated their interplay and plotted a co-expression heatmap (Fig. 7B,C). Finally, we created an interaction diagram that showed the direct interactions among the six genes centered around CHST11 (Fig. 7D).

We selected the top 50 genes from the TCGA database that exhibited either positive or negative correlation with the CHST11 expression levels. An expression heatmap for these genes was then generated (Fig. S5). The GO and KEGG enrichment analyses for CHST11 indicated that CHST11 is primarily localized in the Golgi apparatus in ccRCC cells. It is most significantly associated with processes such as proteoglycan metabolism, GAG biosynthesis, and aminoglycan biosynthesis (Fig. S6).

### CHST11 expression in ccRCC tissues and cells

**Figure 8:**
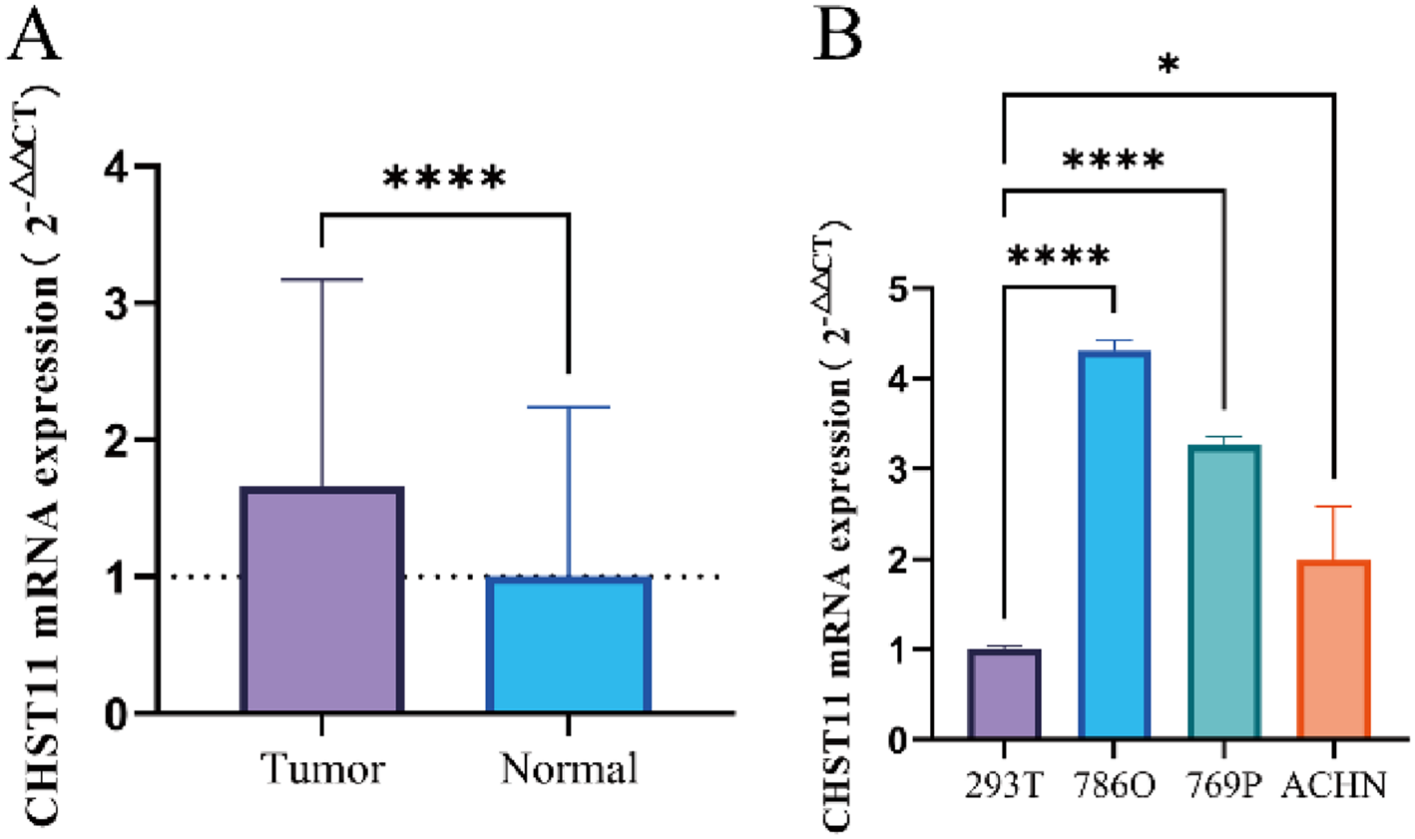
qRT-PCR assessment of the CHST11 mRNA expression levels in ccRCC tissues (**A**) and cell lines (**B**) (*P < 0.05; ****P < 0.0001).

First, we assessed the mRNA expression levels of CHST11 in samples (n = 50) (Table S8) and cells by using qRT-PCR. The results showed that the expression levels of CHST11 were significantly elevated in ccRCC tissues as compared to that in normal tissues (*P* < 0.001) (Fig. 8A). Moreover, the mRNA expression levels of CHST11 in 786O, 769P, and ACHN cells were notably higher than that in 293 T cells (Fig. 8B).

**Figure 9:**
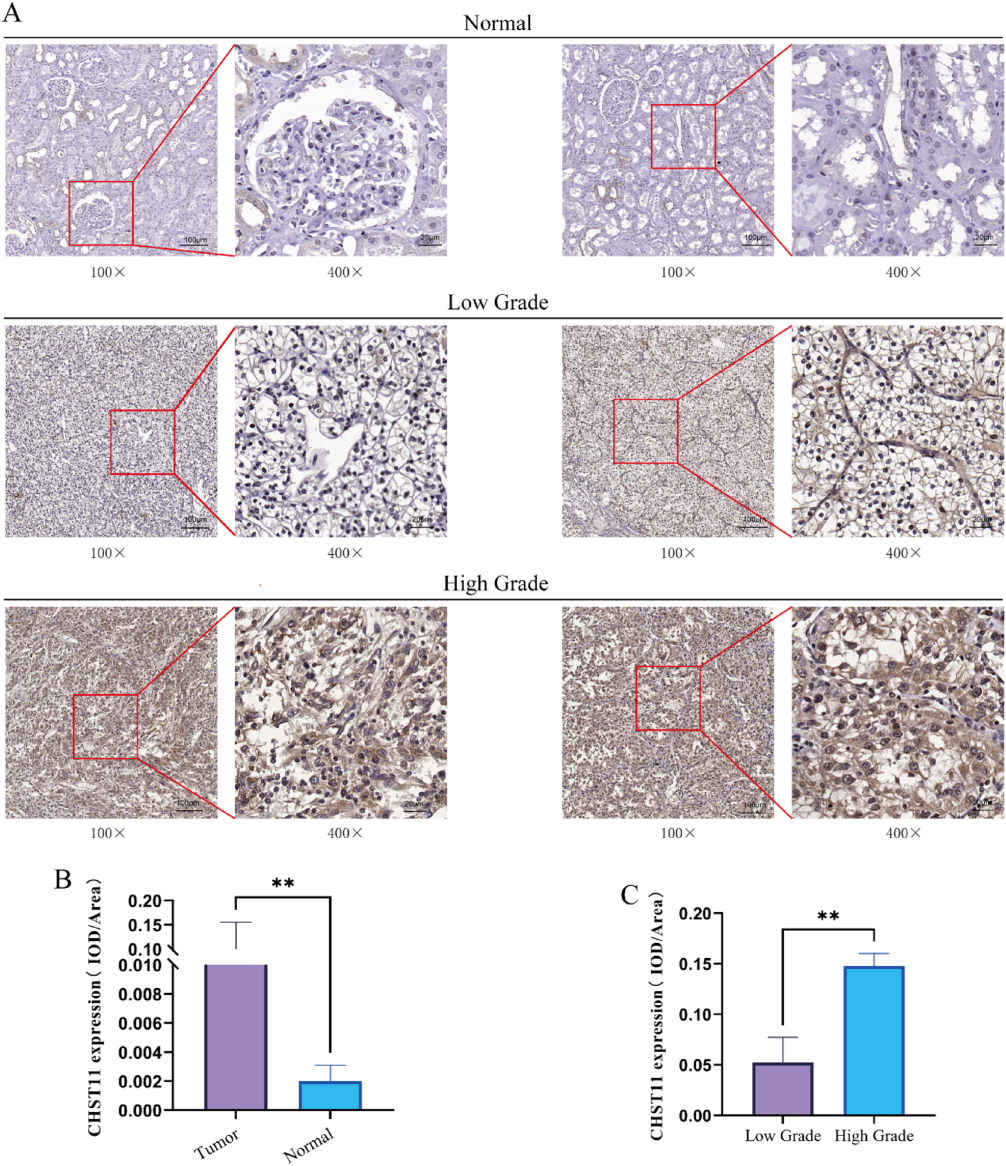
IHC assessment of CHST11 expression in ccRCC. The expression and localization of CHST11in normal tissues and in low-grade and high-grade ccRCC tissues (**A**). Quantitative analysis of CHST11 expression (**B**) and histological malignancy grade (**C**) according to IHC results (**P < 0.01).

We also conducted immunohistochemical staining of both ccRCC and normal tissues. The findings showed that the CHST11 protein primarily localizes in the cell membrane and cytoplasm of ccRCC cells (Fig. 9A). The expression level of the CHST11 protein was significantly higher in ccRCC tissues than in normal tissues (*P* < 0.01) (Fig. 9B). Additionally, the elevated expression of CHST11 was strongly correlated with higher clinical stages of the tumor (*P* < 0.01) (Fig. 9C).

### *CHST11* knockdown inhibited the proliferation, migration, and invasive capabilities of ccRCC cells

**Figure 10:**
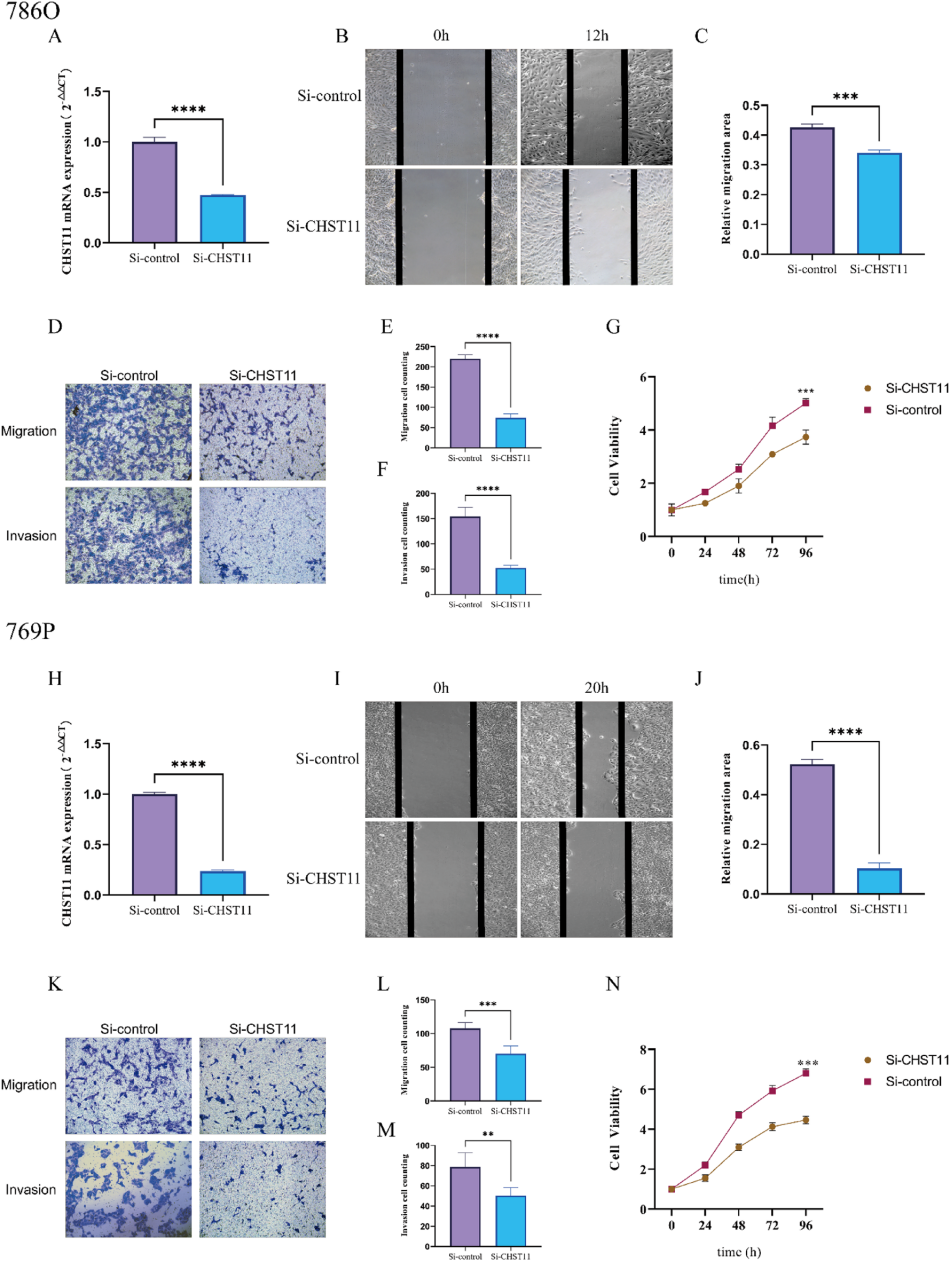
*CHST11* knockdown reduces the malignancy of ccRCC. Assessment of transfection efficiency (**A**,**H**). Wound healing assay (**B**,**I**). Quantitative analysis of the wound healing rate (**C**,**J**). Transwell assay results (**D**,**K**). Quantitative analysis of Transwell assay (**E**,**F**,**L**,**M**). Results of CCK-8 assay (**G**,**N**) (**P < 0.01,***P < 0.001,****P < 0.0001).

To investigate the biological functions of CHST11 in ccRCC cells, we designed siRNA interference agents to knockdown the *CHST11* gene. The transfection efficiency was confirmed by qRT-PCR analyses (Fig. 10A,H).

Wound healing assay confirmed a reduction in the cell migration ability of the si-CHST11 group (Fig. 10B,C,I,J). Transwell assays showed decreased cell migration and invasion capabilities in the si-CHST11 group as compared to that in the si-control group (*P* < 0.01) (Fig. 10D–F,K–M). The results of the CCK-8 assay confirmed reduced cell proliferation in the si-CHST11 group as compared to that in the si-control group (*P* < 0.001) (Fig. 10G,N). These findings indicate that the *CHST11* gene knockdown inhibits the proliferation, migration, and invasive capabilities of ccRCC cells.

### *CHST11* overexpression promoted the proliferation, migration, and invasive capabilities of ccRCC cells

**Figure 11:**
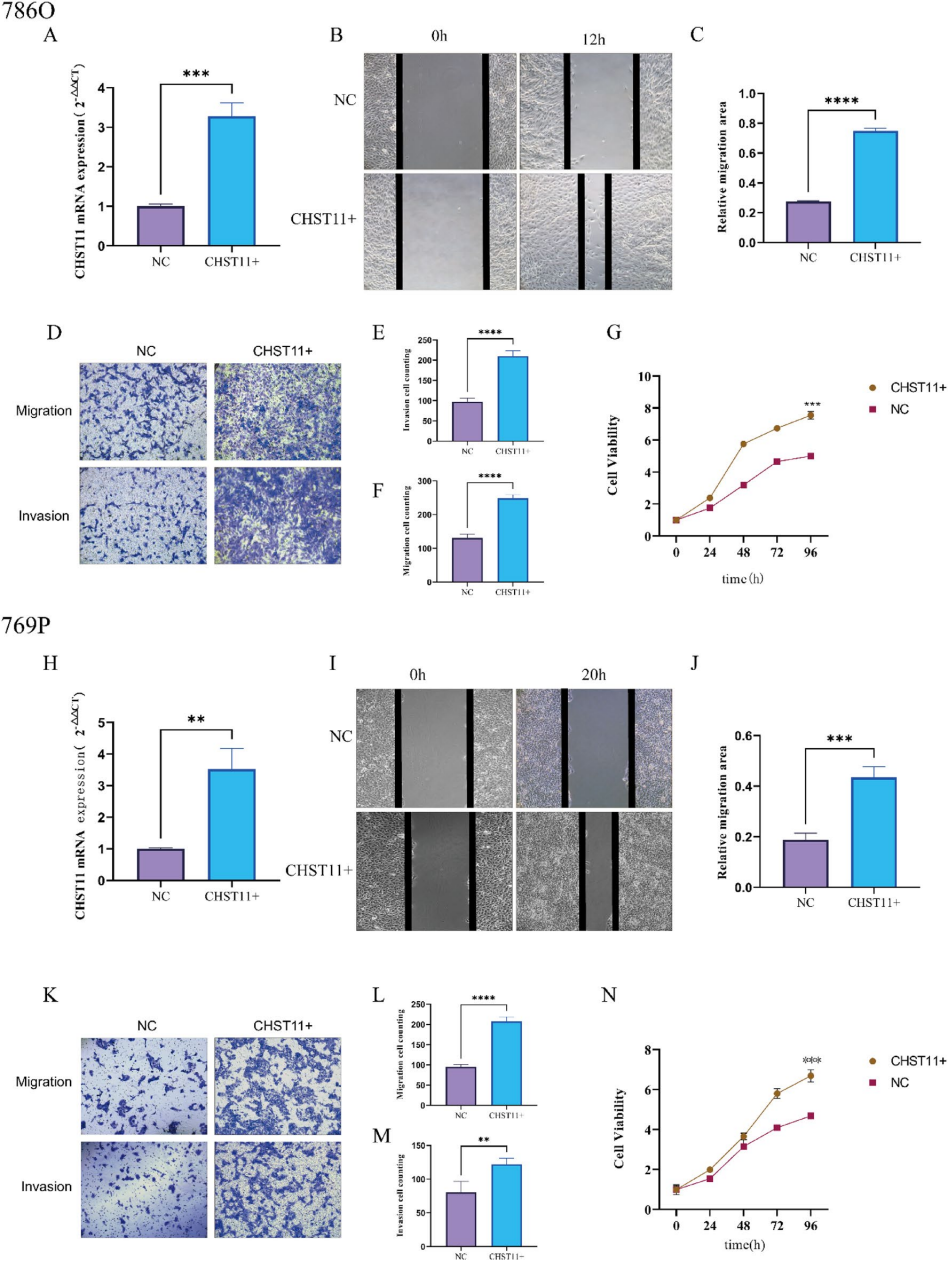
*CHST11* overexpression promotes the malignancy of ccRCC cells. Assessment of transfection efficiency (**A**,**H**). Wound healing assay (**B**,**I**). Quantitative analysis of the wound healing rate (**C**,**J**). Transwell assay results (**D**,**K**). Quantitative analysis of Transwell assay (**E**,**F**,**L**,**M**). CCK-8 assay results (**G**,**N**) (**P < 0.01, ***P < 0.001, ****P < 0.0001).

We constructed an overexpression plasmid for the *CHST11* gene. The plasmid was transfected into 786O and 769P cells by using a transfection reagent. qRT-PCR analyses were then conducted to verify transfection efficiency (Fig. 11A,H).

In 786O and 769P cells, wound healing assay confirmed the enhanced cell migration ability of the CHST11 overexpression group (Fig. 11B,C,I,J). Transwell assays confirmed increased cell migration and invasive capabilities of the CHST11 overexpression group as compared to that of the control group (*P* < 0.01) (Fig. 11D–F,K–M). The results of the CCK-8 assay confirmed enhanced cell proliferation in the CHST11 overexpression group as compared to that in the control group (*P* < 0.001) (Fig. 11G,N). These findings indicate that *CHST11* overexpression enhances the proliferation, migration, and invasive capabilities of ccRCC cells.

## Discussion

ccRCC not only represents an aberrant proliferation of renal cells, but it also indicates a metabolic disorder that manifests as alterations in lipid metabolism. As a metabolic disease, the metabolic pathways in ccRCC cells influence the phenotypic behavior of tumor cells and affect the TME, thereby facilitating the growth of cancer cells^18^. The alterations in these metabolic pathways primarily occur through diverse routes, including glycolysis, amino acid metabolism, oxidative phosphorylation impairment, and lipid metabolism^19^. Moreover, metabolic reprogramming is crucial in the incipient stages of cancer, which augments the malignancy of ccRCC cells^20^. A substantial shift in lipid metabolism occurs in ccRCC cells, accompanied by marked reshaping or anomalous upregulation of lipid proteins in the cellular membrane, thereby facilitating the growth and proliferation of ccRCC cells^21^.

Recent studies have indicated a close correlation between alterations in the surface glycoproteins of ccRCC cells and the metastatic potential of tumors. Glycoproteins participate in the connections between cells and also between cells and the extracellular matrix and thus influence the invasive capabilities of cells^22,23^. Several studies have indicated that GAGs show elevated expression in various cancers and play a role in tumor progression and metastasis. GAGs serve as integral components of proteoglycans and provide structural support to the extracellular matrix (ECM); thus, they mediate cellular behavior by interacting with numerous proteins on the cell surface or within the ECM and regulate cellular processes such as adhesion, migration, proliferation, and differentiation^24,25^. Recent studies suggest that certain GAGs can regulate stem cell differentiation through their interactions with proteins^26,27^.

CHST11 is a critical enzyme in the synthesis of GAGs, particularly CS. According to previous studies, CHST11 is abnormally expressed in various malignant tumors, and it is closely associated with the clinicopathological features of cancer and the prognosis of cancer patients. Li et al.^28^ found that the CHST11 expression level was significantly elevated in lung cancer tissues and correlated with a poor patient prognosis. Relevant studies have indicated that CHST11 may promote the progression of endometrial cancer by activating pathways such as the Wnt signaling pathway and promoting epithelial-mesenchymal transition^29^. CHST11 may promote the metastasis of non-small cell lung cancer cells through dysregulation of ceruloplasmin and intracellular iron balance^8^. However, the role of the *CHST11* gene in ccRCC remains unclear.

Recent studies posit that cancer metabolic reprogramming may interfere with the antitumor immune response. Through Warburg’s effect, cancer cells can intricately influence the generation of the corresponding intermediates in oxidative-reductive reactions, thereby attenuating the proliferation, differentiation, activation, and functionality of immune cells^30^. The aberrant production of metabolites and intermediates in the TME also probably exerts a profound effect on these immune processes.

The tumor immune microenvironment plays a crucial role in tumor progression and prognosis, immune evasion, and immunotherapy. Immune cells can either promote or inhibit the growth and metastasis of tumors^31^. The analysis of tumor immunotherapy involves the activation of immune cells within the body and the enhancement of the organism’s anti-tumor immune response, with the specific aim of eliminating minuscule residual tumor foci, suppressing tumor growth, and using therapeutic strategies to disrupt immune tolerance^32^. Xiong et al.^33^ showed that the *CHST11* gene is upregulated in liver cancer cells, which promoted the infiltration of Treg cells in tumor tissues; thus, correlating with immunosuppressive function. Silencing the *CHST11* gene inhibited cell proliferation and migration. Our results exhibited a high expression of *CHST11*, which is concomitant with a reduction in the infiltration of mesenchymal stem cells, NK T cells, and T-helper cells. In this context, NK cells are regarded as the primary bastion against hematogenous metastatic tumor cells, and reduced NK cell levels may favor an impending metastasis^30^. The decreased NK cell levels frequently coincide with the increased CD47 expression, which is related to a more invasive phenotype and a poor prognosis of ccRCC patients^34^. During ccRCC occurrence, immunoglobulins play a critical role in the evolution of neoplastic cells. Because of the enrichment of proinflammatory cytokines and growth factors in the TME, their sustained presence may paradoxically facilitate cancer progression, leading to uncontrolled malignant proliferative responses^30,35^. It is plausible that CHST11 may mediate the malignant phenotype of ccRCC cells through the modulation of the TME. The immune microenvironment and tumor cells have an intricate relationship. In this context, we posit that *CHST11* expression can enhance their interplay. Hence, these findings will help identify novel indicators or adjunct therapeutic targets to monitor immunotherapy efficacy.

m^6^A, which functions as a genetic regulatory mechanism, enables regulated proteins to drive aberrant transcription, processing, and translation of the target transcripts. This subsequently affects the development of various diseases, including the onset, progression, and prognosis of cancer^36^. m^6^A-associated genes comprise a total of 23 writers, erasers, and readers. The equilibrium among these genes is conducive to maintain the homeostasis of gene expression. Herman et al.^37^ confirmed that the *CHST11* gene is upregulated in breast cancer, with a lower CpG island methylation level in its gene sequence. Higher levels of methylation can silence genes, thereby significantly inhibiting tumor proliferation and differentiation. In the present investigation, we elucidated the intricate relationship between m^6^A regulatory factors and CHST11, along with their prognostic significance in ccRCC cells. The findings revealed a prominent positive correlation between *CHST11* and *RBM15B*, *VIRMA*, *ZC3H13*, *YTHDF3*, *YTHDC2*, and *IGF2BP3*. The aberrant expression of these factors in ccRCC, coupled with their association with *CHST11*, may enable the elucidation of anomalous expression of *CHST11* in patients with ccRCC. We identified m6A-associated genes that exhibit the highest correlation with *CHST11*. We utilized these correlated genes to construct a prognostic model to predict patient outcomes. The methylation of promoter regions also exerts a significant influence on *CHST11* expression. Notably, cg24946597 represents the core methylation region in the *CHST11* gene. Nevertheless, these outcomes necessitate substantiation through the execution of methylation-specific PCR. Overall, the findings of the present study underscore a novel epigenetic pattern of *CHST11*. Moreover, we used the STRING database to construct a PPI network for *CHST11*. The results of functional enrichment analysis further corroborate the involvement of CHST11 in processes such as proteoglycan metabolic processes and GAG biosynthetic processes.

Finally, our study conducted relevant experiments on clinically collected tumor tissues and matched normal tissues. IHC assay was used to provide additional evidence. To determine the intricate biological functions of CHST11 in ccRCC cells, we conducted scratch assays, Transwell experiments, and CCK-8 assays. The deletion of *CHST11* confirmed a decrease in the proliferation, migration, and invasive capabilities of ccRCC cells. The malignancy of ccRCC cells reduced following *CHST11* gene knockdown. Conversely, *CHST11* gene overexpression amplified the proliferation, migration, and invasive capacities of ccRCC cells, indicating an augmentation of cell malignancy. In summary, the results of this study suggest that the *CHST11* gene family may play a crucial role in the occurrence and development of ccRCC. It is also a promising prognostic marker and therapeutic target for ccRCC.

The present study has some limitations. First, a limited number of tissue samples were included, and future studies should include more clinical samples. Further support based on prospective studies and a larger sample size is required. Second, the biological functional studies in this experiment were primarily based on cell experiments, and further studies based on animal experiments are warranted.

## Conclusion

In summary, bioinformatics analysis coupled with experimental evidence indicates a marked upregulation of *CHST11* expression in ccRCC, which correlates with clinicopathological factors, poor prognosis, and the immune microenvironment. For the first time, we suggest that *CHST11* overexpression promotes the proliferation, migration, and invasive capabilities of ccRCC cells. Thus, CHST11 may emerge as a novel biomarker for the therapeutic intervention of ccRCC.

### Supplementary Information

Supplementary Figure S1.

Supplementary Figure S2.

Supplementary Figure S3.

Supplementary Figure S4.

Supplementary Figure S5.

Supplementary Figure S6.

Supplementary Table S1.

Supplementary Table S2.

Supplementary Table S3.

Supplementary Table S4.

Supplementary Table S5.

Supplementary Table S6.

Supplementary Table S7.

Supplementary Table S8.

## Data Availability

The datasets generated during and/or analysed during the current study are available from the corresponding author on reasonable request.
