# Supplementary material for: Effect of CHST11, a novel biomarker, on the biological functionalities of clear cell renal cell carcinoma
Source: Sci Rep. 2024 Apr 2;14:7704. doi: 10.1038/s41598-024-58280-8 (PMC10987617; doi:10.1038/s41598-024-58280-8)
Supplement: Supplementary file 1 — Supplementary Figure S1. [file 41598_2024_58280_MOESM1_ESM.docx]

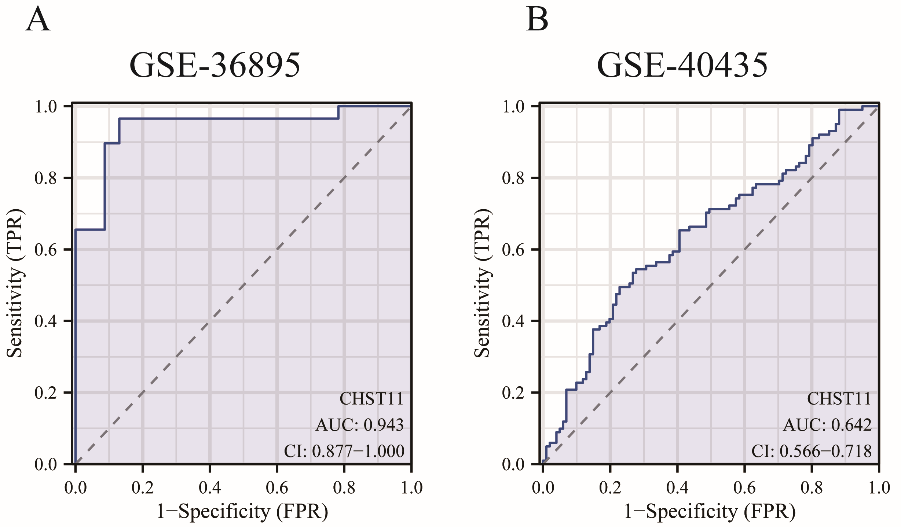


supplementary -Figure S1 Diagnostic Efficacy of CHST11 in GSE36895 and GSE40435. A.ROC curves of CHST11 for diagnosing ccRCC in GSE36895.B. ROC curves of CHST11 for diagnosing ccRCC in GSE40435.
