# Supplementary material for: Effect of CHST11, a novel biomarker, on the biological functionalities of clear cell renal cell carcinoma
Source: Sci Rep. 2024 Apr 2;14:7704. doi: 10.1038/s41598-024-58280-8 (PMC10987617; doi:10.1038/s41598-024-58280-8)
Supplement: Supplementary file 2 — Supplementary Figure S2. [file 41598_2024_58280_MOESM2_ESM.docx]

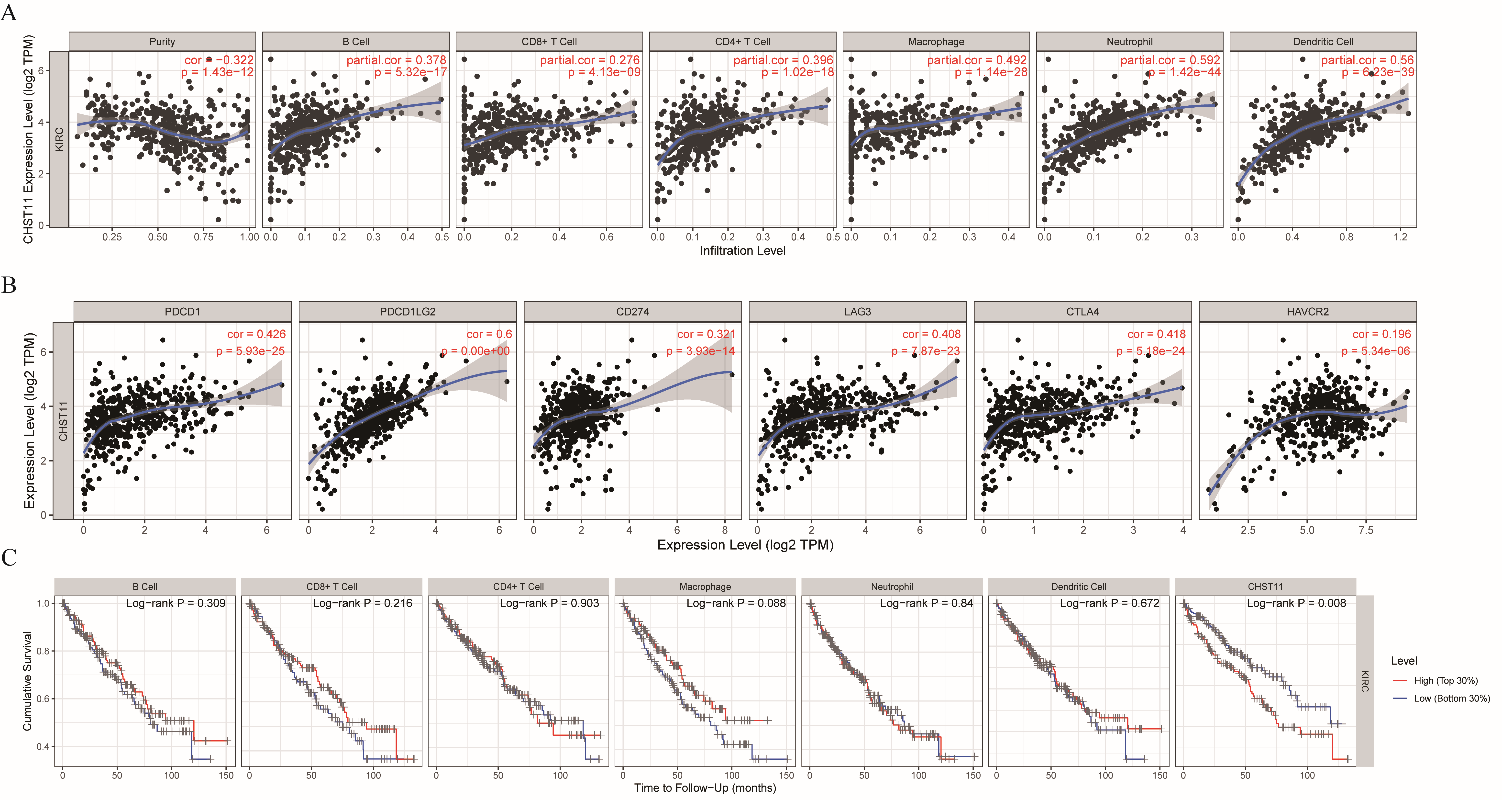


supplementary -Figure S2 Correlation between CHST11 Expression and Immune Infiltration in ccRCC in the TIMER Database.A. Correlation between CHST11 and six immune cell types.B. Correlation between CHST11 and six immune checkpoint genes.C. Survival curves depicting the relationship between CHST11 and six immune cell types.
