# Supplementary material for: Effect of CHST11, a novel biomarker, on the biological functionalities of clear cell renal cell carcinoma
Source: Sci Rep. 2024 Apr 2;14:7704. doi: 10.1038/s41598-024-58280-8 (PMC10987617; doi:10.1038/s41598-024-58280-8)
Supplement: Supplementary file 3 — Supplementary Figure S3. [file 41598_2024_58280_MOESM3_ESM.docx]

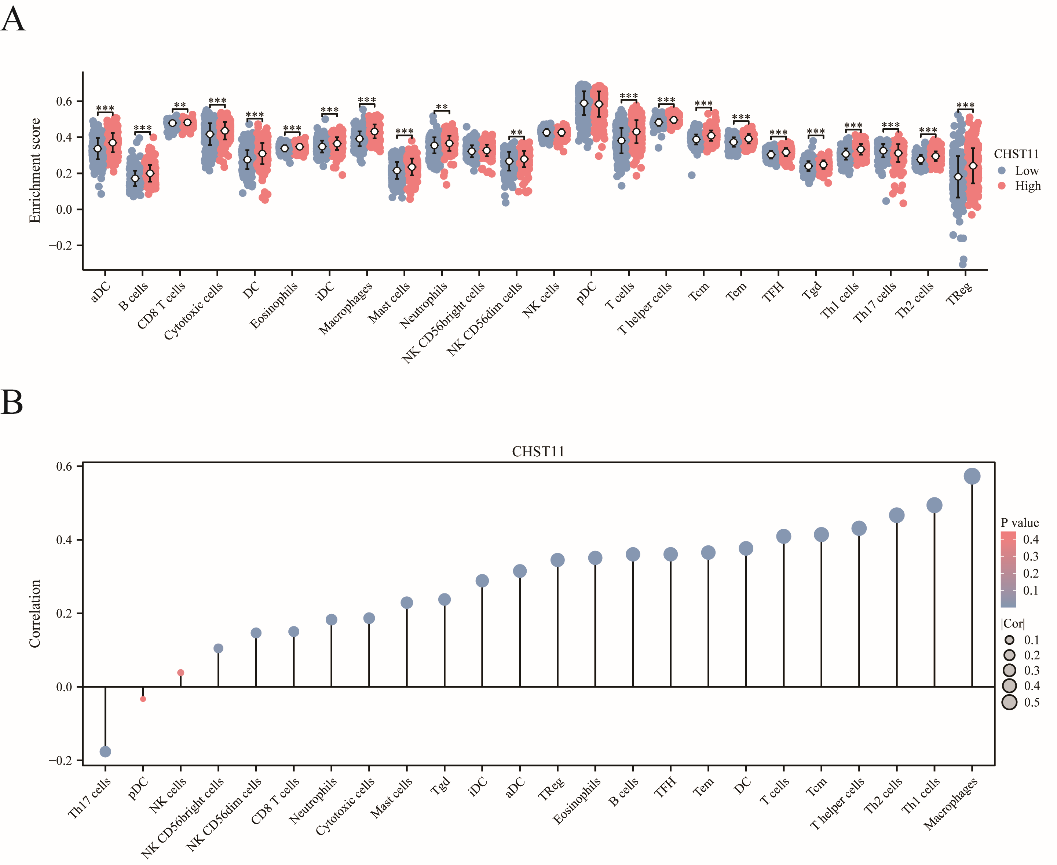


supplementary -Figure S3 Correlation Between CHST11 Expression and Immune Cell Infiltration in ccRCC (ssGSEA).A. Correlation between CHST11 and the infiltration levels of 24 immune cell types.B. Correlation between CHST11 and the infiltration of 24 immune cell types.
