# Supplementary material for: Effect of CHST11, a novel biomarker, on the biological functionalities of clear cell renal cell carcinoma
Source: Sci Rep. 2024 Apr 2;14:7704. doi: 10.1038/s41598-024-58280-8 (PMC10987617; doi:10.1038/s41598-024-58280-8)
Supplement: Supplementary file 4 — Supplementary Figure S4. [file 41598_2024_58280_MOESM4_ESM.docx]

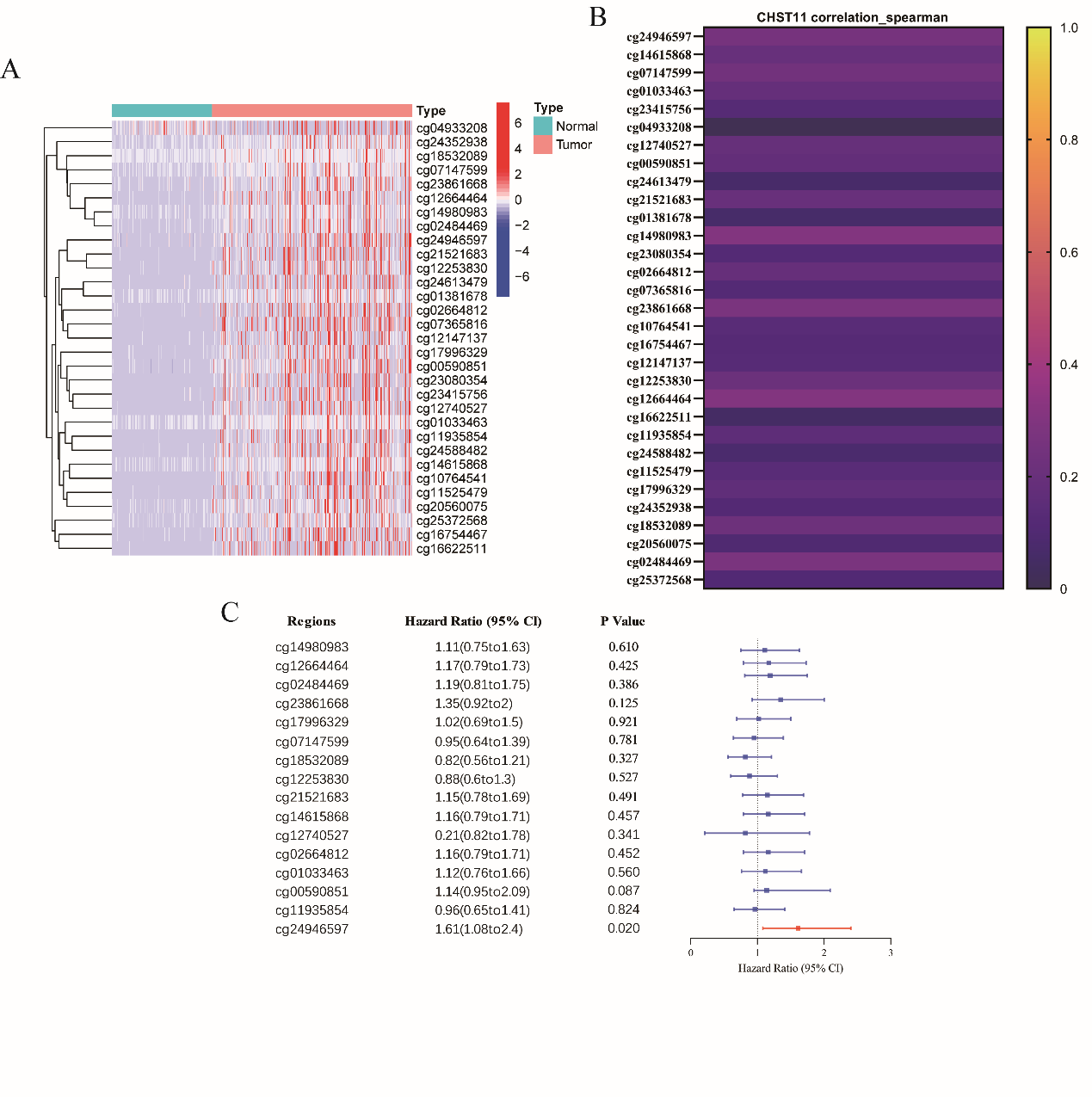
 supplementary -Figure S4 A. Heatmap depicting the correlation between CHST11 promoter region methylation islands and expression in ccRCC.B. Correlation heatmap between CCHST11mRNA expression and CpG region methylation status.C. Forrest plot for CpG island survival analysis.
