# Supplementary material for: Effect of CHST11, a novel biomarker, on the biological functionalities of clear cell renal cell carcinoma
Source: Sci Rep. 2024 Apr 2;14:7704. doi: 10.1038/s41598-024-58280-8 (PMC10987617; doi:10.1038/s41598-024-58280-8)
Supplement: Supplementary file 5 — Supplementary Figure S5. [file 41598_2024_58280_MOESM5_ESM.docx]

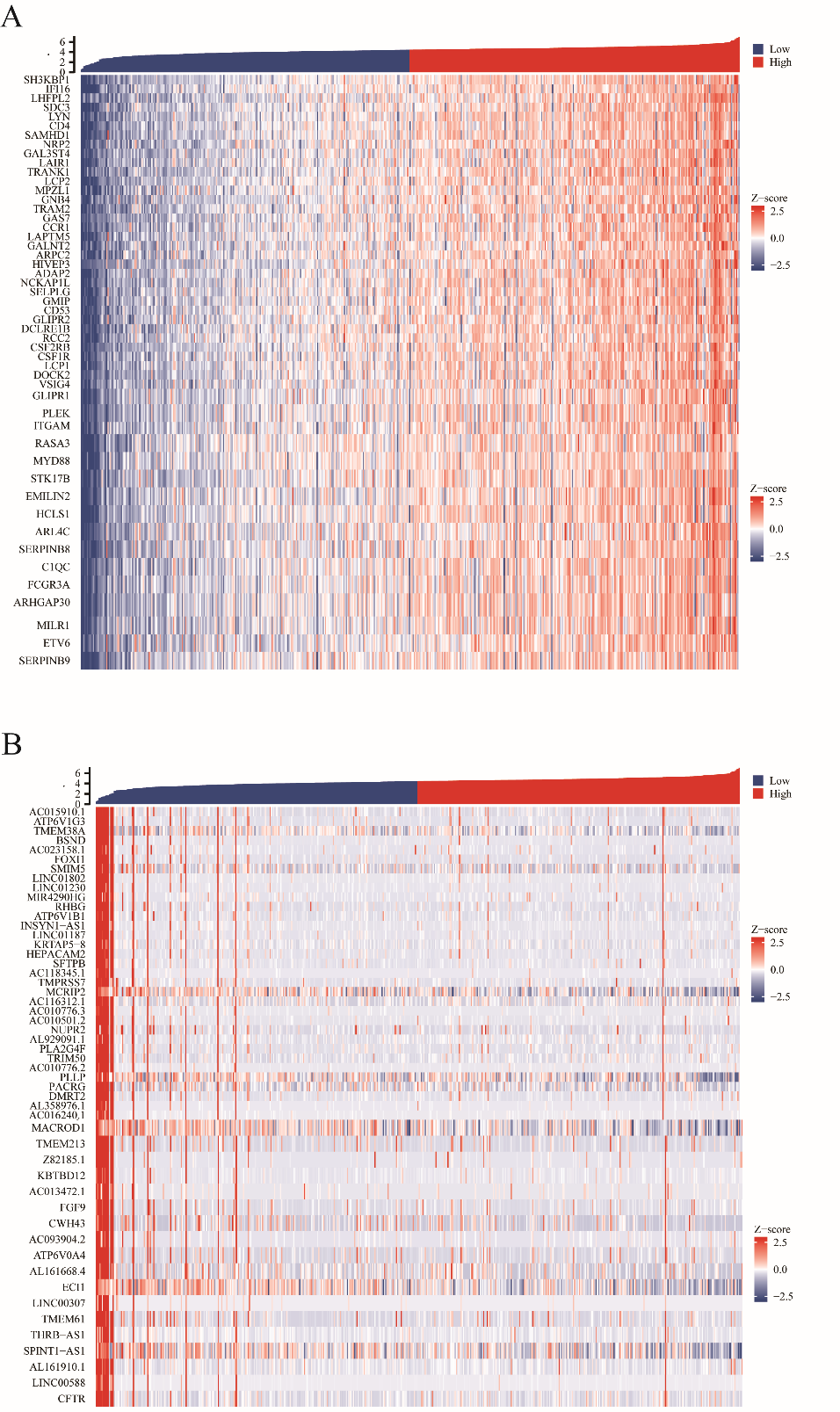


supplementary -Figure S5 A.The top 50 genes that positive correlation with the expression levels of CHST11.B. The top 50 genes that negative correlation with the expression levels of CHST11
