# Supplementary material for: Effect of CHST11, a novel biomarker, on the biological functionalities of clear cell renal cell carcinoma
Source: Sci Rep. 2024 Apr 2;14:7704. doi: 10.1038/s41598-024-58280-8 (PMC10987617; doi:10.1038/s41598-024-58280-8)
Supplement: Supplementary file 6 — Supplementary Figure S6. [file 41598_2024_58280_MOESM6_ESM.docx]

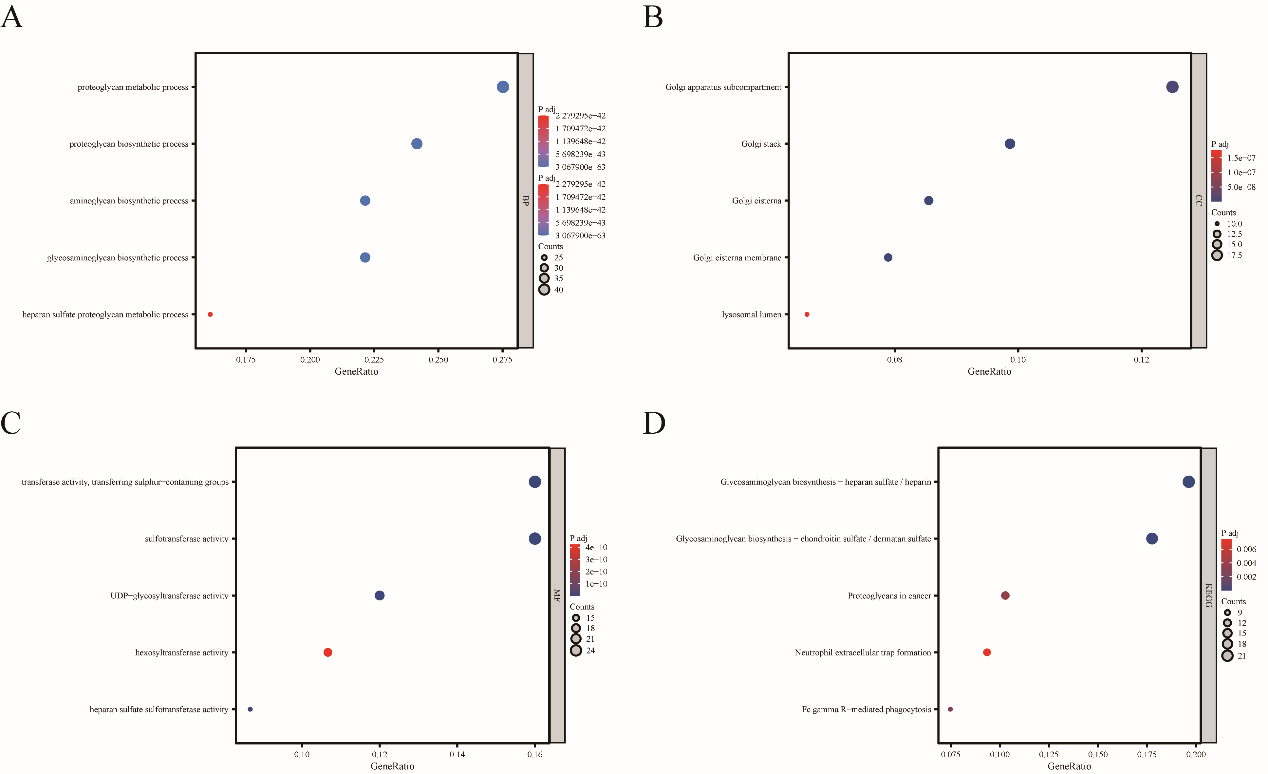


supplementary -Figure S6 GO and Kyoto encyclopedia of genes and genomes (KEGG) pathways（KEGG）enrichment analysis A. BiologicalProcess（BP）.B.Cellular component（CC）.C. Molecular funtion（MF）.D. KEGG)pathways.
