# Supplementary material for: Effect of CHST11, a novel biomarker, on the biological functionalities of clear cell renal cell carcinoma
Source: Sci Rep. 2024 Apr 2;14:7704. doi: 10.1038/s41598-024-58280-8 (PMC10987617; doi:10.1038/s41598-024-58280-8)
Supplement: Supplementary file 7 — Supplementary Table S1. [file 41598_2024_58280_MOESM7_ESM.docx]

supplementary -Table S1 The datasets included in this study that obtained from the GEO database.

| Dataset | ccRCC tissues(n) | Normal tissues(n) | Total tissues(n) | Platform |
| --- | --- | --- | --- | --- |
| GSE53757 | 72 | 72 | 144 | GPL570 |
| GSE40435 | 101 | 101 | 202 | GPL10558 |
| GSE15641 | 32 | 23 | 55 | GPL96 |
| GSE36895 | 29 | 23 | 52 | GPL570 |
| Total | 234 | 219 | 453 | - |
