# Supplementary material for: Effect of CHST11, a novel biomarker, on the biological functionalities of clear cell renal cell carcinoma
Source: Sci Rep. 2024 Apr 2;14:7704. doi: 10.1038/s41598-024-58280-8 (PMC10987617; doi:10.1038/s41598-024-58280-8)
Supplement: Supplementary file 8 — Supplementary Table S2. [file 41598_2024_58280_MOESM8_ESM.docx]

supplementary -Table S2 The correlation between CHST11 mRNA expression and the clinical pathological features of ccRCC patients obtained from the TCGA database.

| Characteristics | Low expression of CHST11 | High expression of CHST11 | P value |
| --- | --- | --- | --- |
| n | 270 | 271 |  |
| Age, n (%) |  |  | 0.966 |
| <= 60 | 134 (24.8%) | 135 (25%) |  |
| > 60 | 136 (25.1%) | 136 (25.1%) |  |
| Gender, n (%) |  |  | 0.054 |
| Female | 104 (19.2%) | 83 (15.3%) |  |
| Male | 166 (30.7%) | 188 (34.8%) |  |
| Pathologic T stage, n (%) |  |  | < 0.001 |
| T1 | 156 (28.8%) | 123 (22.7%) |  |
| T2 | 40 (7.4%) | 31 (5.7%) |  |
| T3 | 73 (13.5%) | 107 (19.8%) |  |
| T4 | 1 (0.2%) | 10 (1.8%) |  |
| Pathologic N stage, n (%) |  |  | 0.017 |
| N0 | 120 (46.5%) | 122 (47.3%) |  |
| N1 | 3 (1.2%) | 13 (5%) |  |
| Pathologic M stage,(%) |  |  | 0.049 |
| M0 | 220 (43.3%) | 209 (41.1%) |  |
| M1 | 31 (6.1%) | 48 (9.4%) |  |
| AJCC stage, (%) |  |  | 0.011 |
| Stage I | 154 (28.6%) | 119 (22.1%) |  |
| Stage II | 31 (5.8%) | 28 (5.2%) |  |
| Stage III | 52 (9.7%) | 71 (13.2%) |  |
| Stage IV | 33 (6.1%) | 50 (9.3%) |  |
| WHO/ISUP, (%) |  |  | < 0.001 |
| G1 | 9 (1.7%) | 5 (0.9%) |  |
| G2 | 135 (25.3%) | 101 (18.9%) |  |
| G3 | 105 (19.7%) | 102 (19.1%) |  |
| G4 | 15 (2.8%) | 61 (11.4%) |  |
| Laterality, n (%) |  |  | 0.666 |
| Left | 124 (23%) | 129 (23.9%) |  |
| Right | 146 (27%) | 141 (26.1%) |  |
