# Supplementary material for: Effect of CHST11, a novel biomarker, on the biological functionalities of clear cell renal cell carcinoma
Source: Sci Rep. 2024 Apr 2;14:7704. doi: 10.1038/s41598-024-58280-8 (PMC10987617; doi:10.1038/s41598-024-58280-8)
Supplement: Supplementary file 9 — Supplementary Table S3. [file 41598_2024_58280_MOESM9_ESM.docx]

supplementary -Table S3 Conducting a logistic regression analysis to examine the association between CHST11 mRNA expression and the clinical pathological features of renal cell carcinoma.

| Characteristics | OR | 95% CI | P value |
| --- | --- | --- | --- |
| Age (> 60 vs. <= 60) | 0.993 | 0.709 - 1.390 | 0.966 |
| Gender (Male vs. Female) | 1.419 | 0.994 - 2.026 | 0.054 |
| Laterality (Right vs. Left) | 0.928 | 0.662 - 1.302 | 0.666 |
| Pathologic T stage (T3-T4 vs. T1-T2) | 2.012 | 1.405 - 2.883 | < 0.001 |
| Pathologic N stage (N1 vs. N0) | 4.262 | 1.185 - 15.336 | 0.026 |
| Pathologic M stage (M1 vs. M0) | 1.630 | 0.999 - 2.660 | 0.051 |
| AJCC stage (III-IV vs. I-II) | 1.792 | 1.260 - 2.547 | 0.001 |
| Histologic grade (G3-G4 vs. G1-G2) | 1.845 | 1.308 - 2.604 | < 0.001 |
