# Supplementary material for: Effect of CHST11, a novel biomarker, on the biological functionalities of clear cell renal cell carcinoma
Source: Sci Rep. 2024 Apr 2;14:7704. doi: 10.1038/s41598-024-58280-8 (PMC10987617; doi:10.1038/s41598-024-58280-8)
Supplement: Supplementary file 10 — Supplementary Table S4. [file 41598_2024_58280_MOESM10_ESM.docx]

supplementary -Table S4 The correlation between CHST11 expression level and tumor lymphocyte.

| Factors | r | p value |  |
| --- | --- | --- | --- |
| Activated CD8 T cell (Act_CD8) | 0.436 | <2.2e-16 |  |
| Central memory CD8 T cell (Tcm_CD8) | 0.468 | <2.2e-16 |  |
| Effector memory CD8 T cell (Tem_CD8) | 0.511 | <2.2e-16 |  |
| Activated CD4 T cell (Act_CD4) | 0.638 | <2.2e-16 |  |
| Central memory CD4 T cell (Tcm_CD4) | 0.534 | <2.2e-16 |  |
| Effector memory CD4 T cell (Tem_CD4) | 0.373 | <2.2e-16 |  |
| T follicular helper cell (Tfh) | 0.673 | <2.2e-16 |  |
| Gamma delta T cell (Tgd) | 0.481 | <2.2e-16 |  |
| Type 1 T helper cell (Th1) | 0.642 | <2.2e-16 |  |
| Type 17 T helper cell (Th17) | 0.162 | <0.001 |  |
| Type 2 T helper cell (Th2) | 0.52 | <2.2e-16 |  |
| Regulatory T cell (Treg) | 0.625 | <2.2e-16 |  |
| Activated B cell (Act_B) | 0.503 | <2.2e-16 |  |
| Immature B cell (Imm_B) | 0.539 | <2.2e-16 |  |
| Memory B cell (Mem_B) | 0.423 | <2.2e-16 |  |
| natural killer cell (NK) | 0.522 | <2.2e-16 |  |
| CD56bright natural killer cell (CD56bright) | 0.328 | <9.7e-16 |  |
| CD56dim natural killer cell (CD56dim) | 0.122 | <0.01 |  |
| Myeloid derived suppressor cell (MDSC) | 0.654 | <2.2e-16 |  |
| Natural killer T cell (NKT) | 0.621 | <2.2e-16 |  |
| Activated dendtritic cell (Act_DC) | 0.576 | <2.2e-16 | |
| Plasmacytoid dendtritic cell (pDC) | 0.282 | <4.21e-11 | |
| Immature dendtritic cell (iDC) | 0.029 | 0.497 |  |
| Macrophage (Macrophage) | 0.63 | <2.2e-16 |  |
| Eosinophi (Eosinophil) | 0.203 | <2.37e-6 | |
| Mast (Mast) | 0.443 | <2.2e-16 | |
| Monocyte (Monocyte) | 0.335 | <2.38e-15 | |
| Neutrophil (Neutrophil) | 0.007 | 0.869 |  |
