# Supplementary material for: Effect of CHST11, a novel biomarker, on the biological functionalities of clear cell renal cell carcinoma
Source: Sci Rep. 2024 Apr 2;14:7704. doi: 10.1038/s41598-024-58280-8 (PMC10987617; doi:10.1038/s41598-024-58280-8)
Supplement: Supplementary file 11 — Supplementary Table S5. [file 41598_2024_58280_MOESM11_ESM.docx]

supplementary -Table S5 The correlation between CHST11 expression level and tumor immunostimulators.

| Factors | r | p value |
| --- | --- | --- |
| C10orf54 | 0.272 | <1.95e-10 |
| CD27 | 0.497 | <2.2e-16 |
| CD276 | 0.411 | <2.2e-16 |
| CD28 | 0.638 | <2.2e-16 |
| CD40 | 0.082 | 0.0596 |
| CD40LG | 0.379 | <2.2e-16 |
| CD48 | 0.47 | <2.2e-16 |
| CD70 | 0.223 | <1.95e-07 |
| CD80 | 0.587 | <2.2e-16 |
| CD86 | 0.582 | <2.2e-16 |
| CXCL12 | 0.174 | <5.53e-05 |
| CXCR4 | 0.385 | <2.2e-16 |
| ENTPD1 | 0.295 | <4.55e-12 |
| HHLA2 | 0.146 | <0.001 |
| ICOS | 0.502 | <2.2e-16 |
| ICOSLG | 0.032 | 0.464 |
| IL2RA | 0.616 | <2.2e-16 |
| IL6 | 0.383 | <2.2e-16 |
| IL6R | -0.051 | 0.243 |
| KLRC1 | 0.347 | <1.72e-16 |
| KLRK1 | 0.387 | <2.2e-16 |
| LTA | 0.508 | <2.2e-16 |
| MICB | 0.516 | <2.2e-16 |
| NT5E | 0.104 | 0.0158 |
| PVR | 0.249 | <6.01e-09 |
| RAET1E | -0.159 | <0.001 |
| TMEM173 | 0.396 | <2.2e-16 |
| TMIGD2 | 0.31 | <2.9e-13 |
| TNFRSF13B | - | - |
| TNFRSF13C | - | - |
| TNFRSF14 | -0.184 | <1.89e-05 |
| TNFRSF17 | 0.434 | <2.2e-16 |
| INFRSF18 | 0.289 | <2.2e-16 |
| TNERSF25 | 0.049 | 0.258 |
| TNFRSF4 | -0.052 | 0.23 |
| TNFRSF8 | 0.469 | <2.2e-16 |
| INFRSF9 | 0.519 | <2.2e-16 |
| TNESF13 | -0.171 | <7.61e-05 |
| TNFSF13B | 0.639 | <2.2e-16 |
| TNFSF14 | 0.433 | <2.2e-16 |
| TNFSF15 | 0.08 | 0.0635 |
| TNESF18 | - | - |
| TNFSF4 | 0.404 | <2.2e-16 |
| TNFSF9 | 0.177 | <4.12e-05 |
| ULBP1 | 0.235 | <4.51e-08 |
