# Supplementary material for: Effect of CHST11, a novel biomarker, on the biological functionalities of clear cell renal cell carcinoma
Source: Sci Rep. 2024 Apr 2;14:7704. doi: 10.1038/s41598-024-58280-8 (PMC10987617; doi:10.1038/s41598-024-58280-8)
Supplement: Supplementary file 12 — Supplementary Table S6. [file 41598_2024_58280_MOESM12_ESM.docx]

supplementary -Table S6 The correlation between CHST11 expression level and tumor immunoinhibitory.

| Factors | r | p value |
| --- | --- | --- |
| ADORAZA | -0.028 | 0.512 |
| BTLA | 0.508 | <2.2e-16 |
| CD160 | 0.053 | 0.222 |
| CD244 | 0.4 | <2.2e-16 |
| CD274 | 0.165 | <0.001 |
| CD96 | 0.578 | <2.2e-16 |
| CSF1R | 0.545 | <2.2e-16 |
| CTLA4 | 0.433 | <2.2e-16 |
| HAVCR2 | 0.157 | <0.001 |
| ID01 | 0.018 | 0.679 |
| IL10 | 0.517 | <0.001 |
| IL10RB | 0.221 | <2.51e-07 |
| KDR | -0.215 | <5.34e-07 |
| KIR2DL1 | - | - |
| KIR2DL3 | - | - |
| LAG3 | 0.46 | <2.2e-16 |
| LGALS9 | 0.538 | <2.2e-16 |
| PDCD1 | 0.485 | <2.2e-16 |
| PDCD1LG2 | 0.536 | <2.2e-16 |
| PVRL2 | 0.1 | 0.0204 |
| TGFB1 | 0.33 | <6.54e-15 |
| TGFBR1 | 0.324 | <2.3e-14 |
| TIGIT | 0.554 | <2.2e-16 |
| VTCN1 | 0.005 | 0.909 |
