# Supplementary material for: Effect of CHST11, a novel biomarker, on the biological functionalities of clear cell renal cell carcinoma
Source: Sci Rep. 2024 Apr 2;14:7704. doi: 10.1038/s41598-024-58280-8 (PMC10987617; doi:10.1038/s41598-024-58280-8)
Supplement: Supplementary file 13 — Supplementary Table S7. [file 41598_2024_58280_MOESM13_ESM.docx]

supplementary -Table S7 The correlation between CHST11 expression level and major histocompatibility complex.

| Factors | r | p value |
| --- | --- | --- |
| B2M | 0.186 | <1.6e-05 |
| HLA-A | 0.188 | <1.21e-05 |
| HLA-B | 0.182 | <2.45e-05 |
| HLA-C | 0.152 | <0.001 |
| HLA-DMA | 0.367 | <2.2e-16 |
| HLA-DMB | 0.447 | <2.2e-16 |
| HLA-DOA | 0.434 | <2.2e-16 |
| HLA-DOB | 0.443 | <2.2e-16 |
| HLA-DPA1 | 0.43 | <2.2e-16 |
| HLA-DPB1 | 0.444 | <2.2e-16 |
| HLA-DQA1 | 0.391 | <2.2e-16 |
| HLA-DOA2 | 0.328 | <1.03e-14 |
| HLA-DOB1 | 0.241 | <1.83e-08 |
| HLA-DRA | 0.468 | <2.2e-16 |
| HLA-DRB1 | 0.319 | <5.93e-14 |
| HLA-E | 0.183 | <2.23e-05 |
| HLA-F | 0.135 | <0.001 |
| HLA-G | 0.204 | <2.18e-06 |
| TAP1 | 0.292 | <7.9e-12 |
| TAP2 | 0.272 | <1.89e-10 |
| ТАРВР | 0.191 | <9.5e-06 |
