# Supplementary material for: Effect of CHST11, a novel biomarker, on the biological functionalities of clear cell renal cell carcinoma
Source: Sci Rep. 2024 Apr 2;14:7704. doi: 10.1038/s41598-024-58280-8 (PMC10987617; doi:10.1038/s41598-024-58280-8)
Supplement: Supplementary file 14 — Supplementary Table S8. [file 41598_2024_58280_MOESM14_ESM.docx]

supplementary -Table S8 Characteristics (number, sex, age, ,TNM TNM staging,histological classification) of patients and healthy controls, whose samples were provided.

| Characteristics | Cancer | Normal |
| --- | --- | --- |
| n | 50 | 50 |
| Age (n) |  |  |
| <= 60 | 31 | 31 |
| > 60 | 19 | 19 |
| Gender (n) |  |  |
| Female | 17 | 17 |
| Male | 33 | 33 |
| Pathologic T stage (n) |  |  |
| T1+T2 | 38 | － |
| T3+T4 | 12 | － |
| Pathologic N stage (n) |  |  |
| N0 | 45 | － |
| N1 | 5 | － |
| Pathologic M stage (n) |  |  |
| M0 | 48 | － |
| M1 | 2 | － |
| Furman grade (n) |  |  |
| G1+G2 | 35 | － |
| G3+G4 | 15 | － |
